# Enrichment of Hard Sweeps on the X Chromosome in *Drosophila melanogaster*

**DOI:** 10.1093/molbev/msac268

**Published:** 2022-12-21

**Authors:** Mariana Harris, Nandita R Garud

**Affiliations:** Department of Computational Medicine, University of California Los Angeles, Los Angeles, CA; Ecology and Evolutionary Biology, University of California Los Angeles, Los Angeles, CA; Department of Human Genetics, University of California, Los Angeles, CA

**Keywords:** hard sweeps, soft sweeps, X chromosome, haplotype homozygosity, adaptation, *Drosophila*

## Abstract

The characteristic properties of the X chromosome, such as male hemizygosity and its unique inheritance pattern, expose it to natural selection in a way that can be different from the autosomes. Here, we investigate the differences in the tempo and mode of adaptation on the X chromosome and autosomes in a population of *Drosophila melanogaster*. Specifically, we test the hypothesis that due to hemizygosity and a lower effective population size on the X, the relative proportion of hard sweeps, which are expected when adaptation is gradual, compared with soft sweeps, which are expected when adaptation is rapid, is greater on the X than on the autosomes. We quantify the incidence of hard versus soft sweeps in North American *D. melanogaster* population genomic data with haplotype homozygosity statistics and find an enrichment of the proportion of hard versus soft sweeps on the X chromosome compared with the autosomes, confirming predictions we make from simulations. Understanding these differences may enable a deeper understanding of how important phenotypes arise as well as the impact of fundamental evolutionary parameters on adaptation, such as dominance, sex-specific selection, and sex-biased demography.

## Introduction

Adaptation on the X chromosome has attracted significant interest from evolutionary biologists because its dynamics seem to be distinct from that of autosomes. The X chromosome is hemizygous in males, increasing the visibility of new mutations to natural selection on the X and thus potentially subject to different evolutionary dynamics than autosomes. The X harbors many essential genes, including genes responsible for speciation (Presgraves 2008; Payseur et al. 2018), fertility (Saifi and Chandra 1999), sexual dimorphism (Rice 1984; Dean and Mank 2014), and brain function (Skuse 2005), as well as several genes that are preferentially expressed in one sex (Lercher et al. 2003; Khil et al. 2004; Prince et al. 2010; Jaquiéry et al. 2013). In the classic model organism *Drosophila melanogaster*, there is evidence of a faster rate of adaptive evolution on the X (Meisel et al. 2012; Meisel and Connallon 2013; Charlesworth et al. 2018) and sexually antagonistic selection acting on the sex chromosomes (Innocenti and Morrow 2010; Dean and Mank 2014; Glaser-Schmitt et al. 2021), revealing crucial differences in adaptation between the X and autosomes. Thus, by studying differences in the tempo and mode of adaptation between the X and autosomes, we may increase our understanding of evolution at a molecular level, particularly in the context of sexual dimorphism, sex-biased demography, speciation, and sex chromosome evolution.

The tempo and mode of adaptation in natural populations more broadly has been long debated. Adaptation can be characterized as gradual or rapid (Hermisson and Pennings 2005, 2017; Pritchard et al. 2010; Messer and Petrov 2013), and its pace depends on the availability of adaptive mutations. When these mutations are absent or rare before the onset of selection, either because the effective population size (*N_e_*), adaptive mutation rate (*μ*_*A*_), or their product (*θ*_*A*_ << 1, where *θ*_*A*_ = 4*N*_*e*_*μ*_*A*_) is small, adaptation is expected to be gradual (Hermisson and Pennings 2005; Pennings and Hermisson 2006a). In such a scenario, selective sweeps are expected to be hard, with a single adaptive mutation rising in frequency, leaving behind characteristic deep dips in diversity in the vicinity of the adaptive locus (Messer and Petrov 2013; Hermisson and Pennings 2017). In contrast, when there is a large input of mutations due to large census population sizes and/or mutation rates (e.g., *θ*_*A*_ > 1; Pennings and Hermisson 2006a), or, standing genetic variation (SGV) is abundant (Hermisson and Pennings 2005), adaptation is expected to be rapid. In such a scenario, selective sweeps are expected to be soft, with multiple adaptive mutations on distinct haplotypes sweeping through the population simultaneously, not necessarily resulting in dips in diversity (Messer and Petrov 2013; Hermisson and Pennings 2017).

In addition, when adaptation proceeds from SGV, the probability of a soft sweep is expected to increase if the dominance of the allele involved shifts from recessive and deleterious to dominant and beneficial in a new environment (Muralidhar and Veller 2022). In this scenario, evolutionary and physiological theories of dominance predict that loss of function mutations are generally recessive while gain of function mutations are generally dominant (Wright 1934; Kacser and Burns 1981; Keightley 1996; Falk 2001). Muralidhar and Veller argue that one example of the dominance shift model occurred at the *Ace* locus in *Drosophila*, which encodes the enzyme acetylcholinesterase that catalyzes the breakdown of the neurotransmitter acetylcholine and has evolved adaptations in response to pesticides (Hoffmann et al. 1992; Fournier and Mutero 1994; Mutero et al. 1994; Menozzi et al. 2004; Karasov et al. 2010). Without pesticides, mutations at the *Ace* locus are deleterious and result in less efficient binding of acetylcholine (Hoffmann et al. 1992; Fournier and Mutero 1994). With pesticides, however, mutations at *Ace* are beneficial because they confer resistance to pesticides (Menozzi et al. 2004; Shi et al. 2004). Previous work in multiple species has shown that the beneficial effect of pesticide resistant alleles is dominant (Bourguet and Raymond 1998; Charlesworth 1998), and that the deleterious effect of such mutations in the absence of pesticides is at least partially recessive (Shi et al. 2004; Labbé et al. 2014; Zhang et al. 2015).

When mutations are slightly deleterious and recessive, their effect on fitness will be initially masked, making it more likely that these mutations can segregate at some low frequency in the population (Phadnis and Fry 2005; Agrawal and Whitlock 2011; Manna et al. 2011). This in turn will increase the number of copies of the variant present in the population when the environmental change occurs, resulting in more distinct haplotypes present in the population at the onset of positive selection. Additionally, with dominance shifts, adaptive mutations in the new environment are expected to be at least partially dominant, and thus are less likely to be lost than if they were still recessive. By this logic, soft sweeps are more likely than hard sweeps when there are dominance than when dominance remains constant across environments (Muralidhar and Veller 2022).

Although soft sweeps have been found to be common on the autosomes (Garud et al. 2015; Schrider and Kern 2017; Brand et al. 2020), should they be equally common on the X? The differences in the inheritance patterns of the X chromosome and the autosomes, as well as the exposure of mutations on the hemizygous X can give rise to differences in the signatures of selection found on the X compared with those from the autosomes. To begin with, the effective population size of the X, *N_eX,_* is usually expected to be lower than that of the autosomes, *N_e_*_Auto_. Particularly, in a population with an equal number of males and females, there are only three X chromosomes per every four autosomes, hence, all else equal, *N_eX_*_=_ ¾ *N_e_*_Auto_. This lower population size can increase the effect of genetic drift and lower the mutational input on the X such that $\theta_{AX}=\;0.75\theta_{A\mathrm{auto}}$ (Vicoso and Charlesworth 2006, 2009). Additionally, recessive deleterious mutations are more likely to be efficiently purged from the X compared with autosomes, resulting in less standing variation that can seed adaptation in environmental shifts (Vicoso and Charlesworth 2006; Charlesworth 2012; Charlesworth et al. 2018). These factors can increase the likelihood of hard sweeps on the X chromosome. However, recessive beneficial mutations on the X may be less prone to becoming lost due to stochastic forces, and thus may counteract the expected increase in likelihood of hard sweeps on the X.

In this study, we examine the relative proportion of hard versus soft sweeps on the X and autosomes using the model organism *D. melanogaster*. To date, although evidence for more rapid evolution on the X has been documented in *D. melanogaster*, the relative proportions of hard versus soft sweeps on the X versus autosomes have not been evaluated with a systematic scan. We focus on *D. melanogaster* because the molecular basis of evolution has already been extensively studied in this organism and there exist several well-documented cases of adaptation across the literature. On the autosomes, three cases of recent adaptation are at the loci *Ace*, *Cyp6g1*, and *CHKov1*, due to resistance to pesticides, DDT, and viruses (Mutero et al. 1994; Daborn et al. 2001; Menozzi et al. 2004; Aminetzach et al. 2005; Karasov et al. 2010; Schmidt et al. 2010; Magwire et al. 2011). These three cases were discovered by empirical means and are all soft sweeps arising from either de novo mutations or SGV. On the X chromosome, the gene *Fezzik* is known to be under positive selection as well (Glaser-Schmitt and Parsch 2018; Glaser-Schmitt et al. 2021), and may experience sexual antagonism. This too was discovered by empirical means, but it is unknown if there is a hard or soft sweep at this locus. To quantify hard and soft sweeps, we used haplotype homozygosity statistics we recently developed (Garud et al. 2015) that are capable of detecting and differentiating both types of sweeps and can recover known positive controls. In previous work, we showed that application of these statistics to the autosomal data in the Drosophila Genetic Reference Panel (DGRP) data set (MacKay et al. 2012), which consists of 205 fully phased genomes from a population in North Carolina, provides evidence for abundant soft sweeps on the autosomes (Garud et al. 2015, 2021). Now, our simulations and application of these same statistics to the X chromosome provide evidence that the X chromosome is enriched for hard versus soft sweeps, relative to the autosomes.

## Results

We first examined the expected prevalence of hard and soft sweeps on the X versus autosomes in simulations with parameters relevant to *D. melanogaster*. To do so, we used the forward in time simulator SLiM 3 (Haller and Messer 2019; Haller et al. 2019), which supports simulations of both autosomal and X chromosome evolution (see Materials and Methods). Next, we examined the incidence of hard and soft sweeps in the DGRP data. To do so, we applied haplotype homozygosity statistics we previously developed for detection of hard and soft selective sweeps.

### Simulations of Hard and Soft Sweeps on the X Versus Autosomes

#### Expected Prevalence of Hard vs. Soft Sweeps as a Function of *θ*_*A*_ and Dominance Coefficient

To understand the expected incidence of hard and soft sweeps on the X versus autosomes, we performed simulations of selection under a wide range of evolutionary scenarios. We varied *θ*_*A*_ given its role in generating hard versus soft sweeps (Pennings and Hermisson 2006a, b; Hermisson and Pennings 2017), where $\theta_{AX}=\;0.75\theta_{A\mathrm{auto}}$. We also varied dominance (*h*) given differences in hemizygosity on the X versus autosome, as well as its recently discovered role in generating hard versus soft sweeps (Muralidhar and Veller 2022). We defined the softness of a sweep by the number of distinct mutational origins at the locus under selection at the time of fixation in a sample of *n* = 100 haplotypes, matching the sample size of the DGRP data (Materials and Methods). A simulation was classified as a soft sweep if the sampled population had more than one mutational origin and as a hard sweep if it had a single origin. Finally, because forward in time simulators are computationally intensive when simulating large populations such as *D. melanogaster* (*N_e_* ∼ 1e6), we rescaled the simulation parameters, as described in the Materials and Methods.

In agreement with theoretical expectations (Hermisson and Pennings 2005; Pennings and Hermisson 2006a), figure 1 shows that the number of origins of a sweep increase with *θ*_*A*_ on both the X and the autosomes. Although sweeps on autosomes typically have a higher number of origins compared with the X, this difference depends on the dominance coefficient of mutations. When *h* = 0, selective sweeps are softer on the X than on the autosomes due to a higher chance of recessive beneficial mutations escaping loss on the X compared with autosomes (Charlesworth 1992; Orr and Betancourt 2001). As *h* increases, the softness of sweeps on both the autosomes and the X chromosome increases, though sweeps are softer on the autosomes compared with the X. Moreover, the average number of generations that it takes for a sweep to reach fixation on the X is lower than that observed on the autosomes when *h <* 0.5 but higher when *h* > 0.5 (supplementary fig. S1*A*, Supplementary Material online). This is consistent with the Faster-X theory (Charlesworth et al. 1987; Betancourt et al. 2004; Meisel and Connallon 2013) and with the fact that when adaptation is gradual, sweeps are expected to be hard, but when adaptation is rapid, sweeps are expected to be soft.

**Fig. 1.**
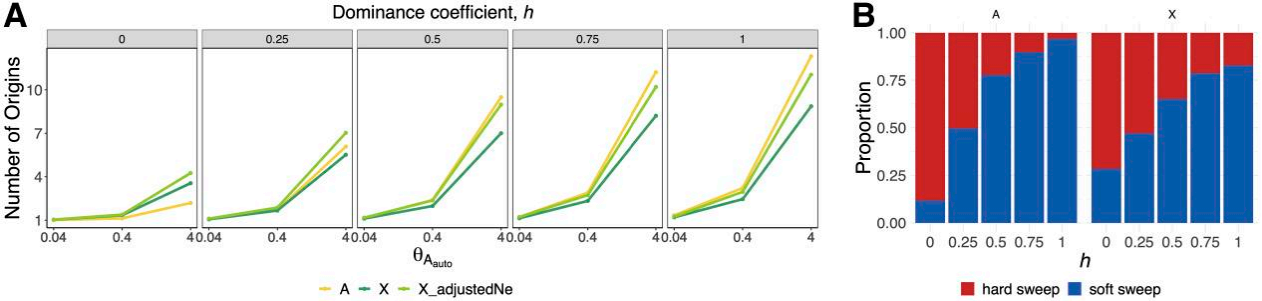
Number of origins as a function of *θ*_*A*_ and dominance coefficient. (*A*) Average number of distinct mutational origins in simulations of selective sweeps from recurrent mutations for $\theta_{A_{\mathrm{auto}}}=0.04$, 0.4, and 4 with *N_e_s* =100. The yellow line represents the number of origins for sweeps on the autosome, whereas the dark green represents the number of origins for sweeps on the X chromosome, in which $\theta_{AX}\;=0.75\theta_{A\mathrm{auto}}\;$. The light green line corresponds to simulations in which *N_eX_* was adjusted such that $\theta_{A_{X}}=\theta_{A_{\mathrm{auto}}}$. Each panel corresponds to a different dominance coefficient, with *h* > 0.5 indicating dominant or partially dominant mutations and *h* < 0.5 recessive or partially recessive mutations. (*B*) Proportion of simulations resulting in hard (red) or soft (blue) sweeps for $\theta_{A_{\mathrm{auto}}}=0.4.\;$ For each combination of parameters, we ran 1,000 simulations. Supplementary Figure S2*A*, Supplementary Material online shows the expected frequency of hard versus soft sweeps arising from SGV on the X versus autosome.

To assess the relative contribution of hemizygosity versus lower *θ*_*A*_ in generating harder sweeps on the X, we adjusted *N_eX_* such that $\theta_{AX}=\;\theta_{A\mathrm{auto}}$ (light green line in fig. 1). In this scenario, the average number of origins increases compared with the uncorrected *N_eX_* (dark green), as the higher *N_eX_* increases the mutational input and consequently, the probability of soft sweeps. Nonetheless, sweeps on the autosomes appear softer than on the X even with the adjusted *N_eX_* for *h* > 0.5, indicating that hemizygosity contributes to harder sweeps on the X. In sum, selective sweeps are more likely to be hard on the X chromosome than on the autosomes due to a combination of lower *θ*_*A*_ and hemizygosity.

In addition to modeling selective sweeps from de novo mutations, we simulated sweeps from SGV. In this scenario, a single mutation is introduced and can segregate and recombine onto multiple haplotypes (Materials and Methods). When there are multiple distinct haplotypes bearing this mutation at the onset of positive selection, this is akin to a soft sweep arising from multiple distinct origins like in the de novo case. However, defining hard and soft sweeps from such simulations is challenging because SLiM only keeps track of the number of distinct origins and not the number of unique haplotypes a mutation may be on at the onset of selection. Therefore, to investigate differences in the softness of sweeps on the X versus autosome in the SGV scenario, we computed the number of distinct haplotypes in a sample of 100 haplotypes when selection ceased (Materials and Methods). This serves as a rough proxy for the softness of a sweep, in which more distinct haplotypes are expected under a soft sweep when compared with a hard sweep. We found that sweeps on the X have a lower number of haplotypes, suggesting harder sweeps, across all values of *h* and starting partial frequencies (PF; supplementary fig. S2*A*, Supplementary Material online). The difference in the number haplotypes is greatest for the completely recessive (*h* = 0) and completely dominant (*h* = 1) scenarios and smallest for additive mutations (*h* = 0.5).

#### Expected Prevalence of Hard Versus Soft Sweeps as a Function of Dominance Shifts

Recently, Muralidhar and Veller showed that dominance shifts increase the likelihood of soft sweeps on the autosomes and hypothesized that this model would lead to harder sweeps on the X compared with autosomes due to the hemizygous state of the X in males. We test this hypothesis following the simulation strategy in Muralidhar and Veller (2022).

In this scenario, mutations at the locus of interest are initially deleterious with selection coefficient *N_e_s_d_* = −100 and dominance *h_d_*. These mutations arise at a rate of *θ*_del_ = 4*N*_*e*_*μ*_del_, where *μ*_del_ is the deleterious mutation rate. After 10*N_e_* generations, the deleterious mutations segregating in the population, if any, become beneficial with selection coefficient *N_e_s_b_* = 100 and dominance *h_b_*. After this, the simulations run until fixation of the beneficial mutation or until the sweep is lost. Following this approach, for each set of parameter values (*s_d_*, *h_d_*, *s_b_*, *h_b_*, *θ*_del_ ), we recorded the proportion of simulations that resulted in no standing variation, lost sweep, hard sweep, and soft sweep. No standing variation refers to the case in which there are no copies of the deleterious variant in the population at the time of the environmental change and lost sweep refers to the case in which mutations are lost after they become beneficial (Materials and Methods). In addition to this multiple origin sweeps scenario, we modeled single origin sweeps from SGV, where an adaptive mutation sweeping through the population emerges from a single common ancestor that was deleterious prior to the onset of positive selection. Here we also vary dominance over time.

**Fig. 2.**
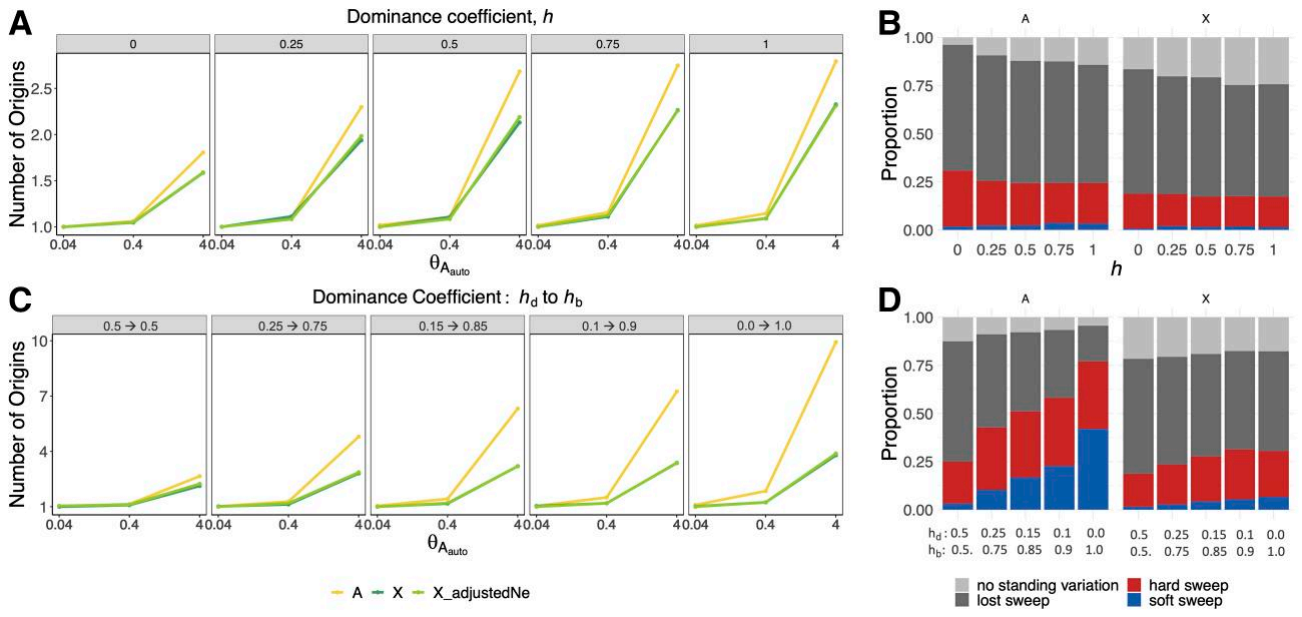
Dominance shifts increase the proportion of soft sweeps on autosomes to a greater extent than on the X. The number of origins on the autosomes and the X for *θ*_*A*_ = 0.04, 0.4, and 4 and different dominance coefficients when dominance is constant (*h*) (*A*) or changing (*h_d_* to *h_b_* before and after the onset of selection, respectively) (*C*). (*B*) The proportion of hard and soft selective sweeps on the autosomes and the X for different dominance coefficients in a model of adaptation to a change in the environment with constant dominance. (*D*) The proportion of hard and soft selective sweeps in the case of dominance shifts. For each combination of parameters, we ran a total of 2,000 simulations of a constant scaled *N*_eAuto_ = 1e6 model, with *N_e_s_d_* and *N_e_s_b_* = 100, $\theta_{\mathrm{de}l_{\mathrm{auto}}}=0.4$, and $\theta_{\mathrm{de}l_{X}}=0.75\theta_{\mathrm{de}l_{\mathrm{auto}}}$. Supplementary Figure S2*B*and*C*, Supplementary Material online shows similar results for sweeps from SGV.

Figure 2 and supplementary figure S2*B*and*C*, Supplementary Material online show that selective sweeps are softer on the autosomes than on the X, irrespective of whether or not there is a dominance shift. When there are dominance shifts, in agreement with previous results (Muralidhar and Veller 2022), the probability of soft sweeps on the autosomes increases with larger shifts, resulting in even larger differences between the number of origins between the X and autosomes. Interestingly, soft sweeps increase in probability not at the expense of hard sweeps, but at the expense of lost sweeps (fig. 2*D*).

The increased probability of hard sweeps on the X is likely driven by more efficient purging of deleterious variation due to stronger genetic drift given a lower *N_eX_* or hemizygosity (Pool and Nielsen 2007; Vicoso and Charlesworth 2009; Charlesworth 2012). To test the effect of lower *N_eX_*, we adjust *N_eX_* to match *N_e_*_Auto_ and find little difference with the uncorrected *N_eX_* case, suggesting that hemizygosity plays a major role in driving hard sweeps on the X. To test the effect of purging on increased hard sweeps on the X, we contrasted simulations in figure 2 with that of supplementary figure S3, Supplementary Material online, in which s*_d_* = 0 (SGVs do not have a deleterious selective coefficient prior to the onset of positive selection). In this scenario, there is a smaller difference in the number of origins between autosomes and the X, indicating that purging of deleterious variants plays an important role in driving harder sweeps on the X. Supporting this notion, the proportion of sweeps for which there is no SGV at the time of the environmental change is larger on the X than on the autosomes for all values of *h* (light gray region in fig. 2*B*and*D*). Additionally, the purging of deleterious mutations overall reduces the number of origins in figure 2*A* when compared with figure 1, where mutations are always beneficial and thus become less likely to get lost as dominance increases.

To tease apart the effect of *h_b_* versus *h_d_* on the softness of a sweep, we simulated a scenario where we hold either *h_b_* constant (*h_b_* = 0.5) and vary *h_d_*, or vice versa. The dominance coefficient before the environmental shift (*h_d_*) influences the probability of loss and consequently the available SGV at the time of the shift. Thus, when *h_b_* = 0.5 and *h_d_* is low (≤0.5), softer sweeps are observed on the autosomes due to deleterious variants being able to persist in the population. After the shift, dominance (*h_b_*) has an effect on the probability that the now beneficial mutations will escape loss and successfully sweep through the population. Thus, when *h_d_* = 0.5 and *h_d_* is high (≥0.5), the proportion of lost sweeps decreases more on the autosomes than on the X, leading to softer sweeps on the autosomes (supplementary fig. S4*C*and*D*, Supplementary Material online).

Finally, we tested the effect of recurrent mutations continuing to enter the population even after a sweep proceeds from SGV. Consistent with Muralidhar and Veller, when recurrent mutations continue to arise after the environmental shift (supplementary fig. S5*A*and*C*, Supplementary Material online), dominance shifts increase the probability that a sweep will contain haplotypes from SGV on both the X and the autosomes. In this scenario, there continues to be a higher proportion of hard sweeps on the X compared with the autosomes (supplementary fig. S5*B*, Supplementary Material online). In summary, these observations indicate that selective sweeps are more likely to be hard on the X chromosome than on the autosomes in changing environments, with or without dominance shifts.

#### Expected Prevalence of Hard Versus Soft Sweeps as a Function of Sexual Antagonism

We investigated the effect of sexual antagonism, whereby a mutation can be beneficial for one sex but harmful for the other, as this has been shown to be a common evolutionary force in *D. melanogaster* (Long and Rice 2007; Innocenti and Morrow 2010; Ruzicka et al. 2019; Glaser-Schmitt et al. 2021). The unique inheritance patterns of the X chromosome can lead sex-dependent selection to act differently between the autosomes and the X. To begin with, autosomes spend equal amounts of time in both sexes, balancing out sexually antagonistic forces, whereas the X chromosome spends two-third of its evolutionary time in females and one-third in males. This could potentially bias selection on the X to be more favorable for females. However, because of male hemizygosity on the X, selection could also favor mutations that benefit males (Rice 1984; Charlesworth et al. 1987; Vicoso and Charlesworth 2006; Frank and Patten 2020). Evidence of sexual antagonism influencing genetic variation has been documented in a range of species including humans (Lercher et al. 2003), aphids (Jaquiéry et al. 2013), mice (Khil et al. 2004), and *Drosophila* (Rice 1992; Long and Rice 2007; Innocenti and Morrow 2010; Prasad et al. 2015; Glaser-Schmitt and Parsch 2018; Ruzicka et al. 2019). However, to our knowledge, the influence of sexually antagonistic selection on the prevalence of hard and soft selective sweeps is not well known. Through simulations, we explored how these forces can influence the signatures of selection on the autosomes and the X chromosome.

**Fig. 3.**
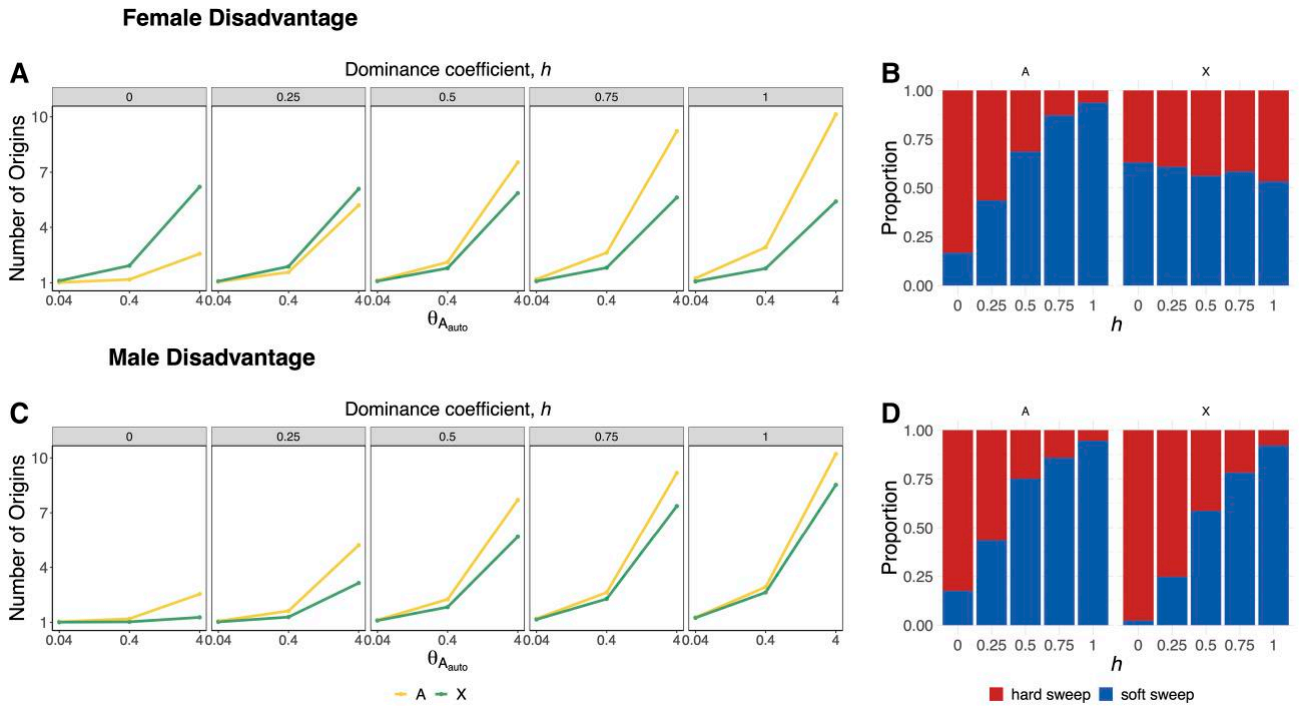
Prevalence of hard and soft selective sweeps for sexually antagonistic selection as a function of *θ*_*A*_ and dominance. In (*A*) and (*B*), mutations are harmful for females and beneficial in males. Selective sweeps are harder on the X except for the case of strongly recessive mutations (∼*h* < 0.25). In (*C*) and (*D*) the mutations are deleterious in males but beneficial for females. In this scenario, the simulated sweeps are harder on the X for all values of dominance. We ran 1,000 simulations for constant scaled *N_e_*_Auto_ = 106 model with $\theta_{\mathrm{de}l_{\mathrm{auto}}}=0.04$, 0.4, and 4 and $\theta_{\mathrm{de}l_{X}}=0.75\theta_{\mathrm{de}l_{\mathrm{Auto}}}$, *s_b_* = 0.01, and *s_d_* = −0.001.

We simulated two scenarios of sexual antagonism: female disadvantage with male advantage and male disadvantage with female advantage. To do this, we introduced sexually antagonistic mutations to the population according to parameter *θ*_*A*_ with a beneficial selection coefficient *s*_*b*_ in one sex and a deleterious selection coefficient *s*_*d*_ in the other sex. We set *s*_*d*_ = −*ks*_*b*_, where *k* is a scalar that modulates the deleterious strength of selection and was set to 0.1. In figure 3, we show the number of origins as a function of *θ*_*A*_ and dominance in a scenario where the introduced mutations are deleterious in females but beneficial in males (fig. 3*A*and*B*) and a scenario where mutations are deleterious in males but beneficial in females (fig. 3*C*and*D*). In the case of female disadvantage, there is a higher average number of origins on the X when mutations are highly recessive (∼*h* < 0.25), otherwise the number of origins is lower on the X than on the autosomes. In the case of male disadvantage, there are a lower number of origins on the X for all values of dominance. These observations suggest that under a model of sexual antagonism, selective sweeps are more likely to be harder on the X chromosome than on the autosomes with the exception of recessive mutations that are female deleterious.

### Analysis of DGRP Data

The evolutionary scenarios explored in simulations in the previous section demonstrate that the expected number of origins for selective sweeps on the X chromosome is generally lower than that of autosomes. Next, we examined the prevalence of hard and soft sweeps on the X and autosomes in the DGRP data set (MacKay et al. 2012), composed of 205 inbred *D. melanogaster* genomes from North America.

#### Diversity on the X Versus Autosomes of *D. melanogaster*

First, we reassessed estimates of the number of segregating sites per base pair (*S*/bp) and nucleotide diversity per base pair (*π*/bp) on the X versus autosomes in two populations of *D. melanogaster*: a derived North American population (DGRP; MacKay et al. 2012) and an ancestral Zambian population (DPGP3; Lack et al. 2015). A previous study argued that the diversity patterns observed in ancestral and derived genomic data could not be explained by neutral demography alone and proposed a model with a 7:1 female biased ancestral sex ratio combined with a population bottleneck that retained this bias along with higher rates of positive selection on the X chromosome in the derived population (Singh et al. 2007).

**Fig. 4.**
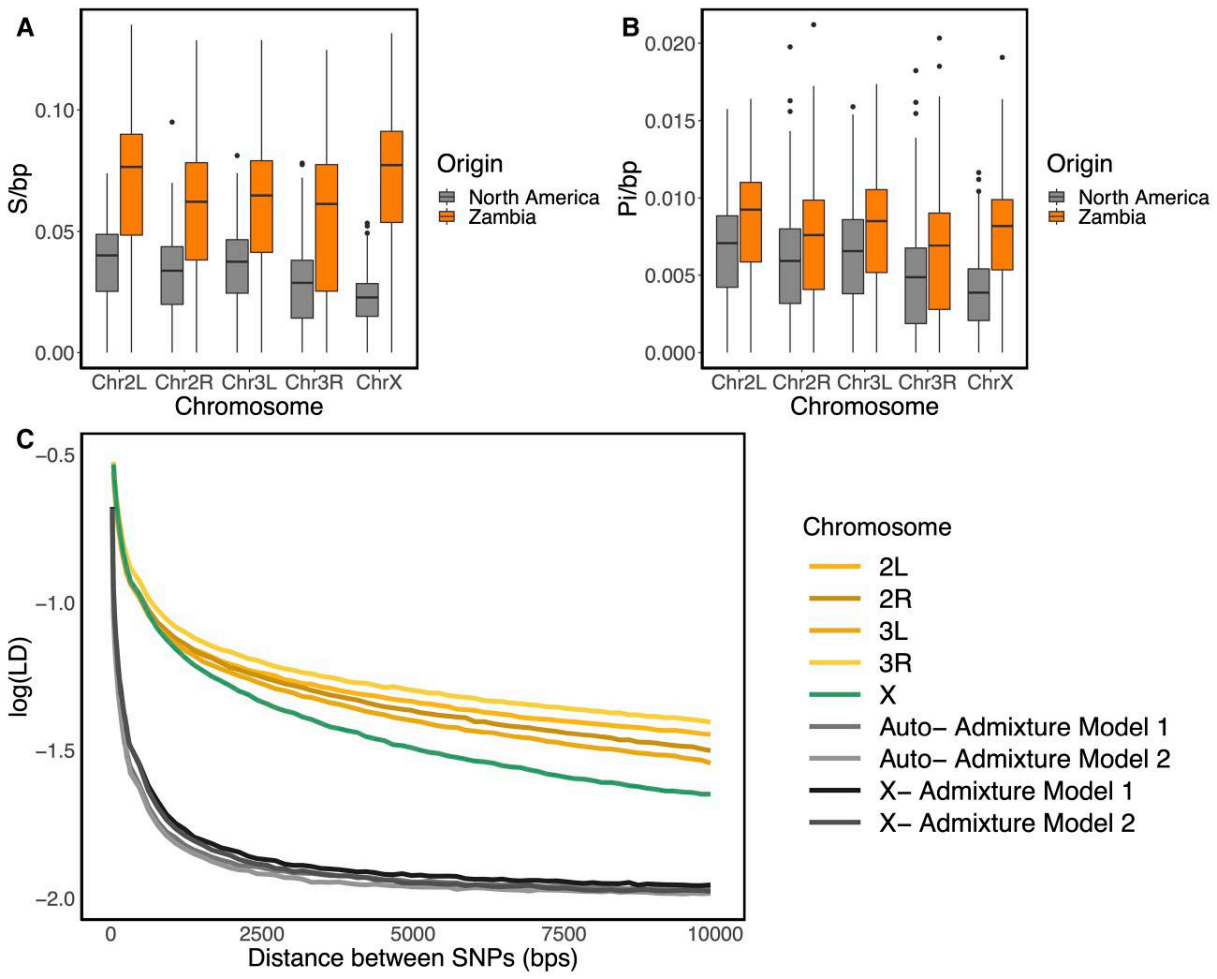
Genetic diversity on the X and autosomes in *D. melanogaster*. *S*/bp (*A*) and *π*/bp (*B*) in the North American (gray) and Zambian (orange) populations with sample size *n* = 100. (*C*) Pairwise LD in North American data and in neutral admixture Models 1 and 2. Regions with low recombination (*ρ* ≤ 5 × 10^−7^ cM/bp) were excluded from LD computations. Demographic models were simulated with *ρ* = 5 × 10^−7^ cM/bp. Low recombination rates are expected to result in higher values of LD and homozygosity. Thus *ρ* = 5 × 10^−7^ cM/bp is conservative for identifying selective sweeps from data (see Materials and methods).

In consonance with the previous findings (Kauer et al. 2002; Dieringer et al. 2005; Thornton and Andolfatto 2006; Pool et al. 2012), genome-wide diversity is significantly reduced in North America relative to Zambia (*P* < 2.2e-16 one-sided Wilcoxon rank sum test for both for *S*/bp and *π*/bp; fig. 4*A*), with a more extreme reduction in diversity on the X compared with the autosomes. Based on the average *S*/bp, we obtain $\theta_{\mathrm{Americ}a_{\mathrm{Auto}}}/\theta_{\mathrm{Zambi}a_{\mathrm{Auto}}}$ = 0.54 (95% confidence interval 0.44–0.64) for autosomal loci and $\;\theta_{\mathrm{Americ}a_{X}}/\theta_{\mathrm{Zambi}a_{X}}$ = 0.31 (0.21–0.41) for the X chromosome. Moreover, *S*/bp and *π*/bp are significantly reduced on the X chromosome relative to the autosomes in the North American population (*P* < 2.2e-16, one-sided Wilcoxon rank sum test for both *S*/bp and *π*/bp; fig. 4*A*), whereas in the ancestral Zambian population, there is no evidence to support a decrease in X chromosome diversity (one-sided Wilcoxon rank sum test *P* = 1 and *P* = 0.95 for *S*/bp and *π*/bp, respectively; fig. 4*A*).

To further explore the role of neutral versus selective forces in generating the observed diversity patterns, we next compared levels of linkage disequilibrium (LD) in the North American population with that of two neutral admixture models previously fit to the data (Duchen et al. 2013; Garud et al. 2021). These demographic models are variations of the Duchen et al. (2013) admixture model and have been shown to fit the DGRP data in terms of multiple summary statistics (Garud et al. 2021). We refer to these models as Models 1 and 2, where in Model 1 the contemporary North American population size is *N_e_*_Am_ = 1,110,000 and in Model 2 *N_e_*_Am_ = 15,984,500. The remaining parameters remain the same across the models and are specified in supplementary table S1, Supplementary Material online and in (Garud et al. 2021).

LD in the DGRP data is elevated compared with neutral expectations generated by Models 1 and 2 for both the autosomes and X chromosome (fig. 4*B*). The elevation of LD on the X is notable given overall higher average recombination rates on the X compared with the autosomes (supplementary fig. S6, Supplementary Material online; Pool et al. 2012). Higher recombination rates on the X in *Drosophila* are due to (1) a higher cM/bp rate on the X versus the autosomes in females (Comeron et al. 2012; Pool et al. 2012), and (2) a greater fraction of X chromosomes than of autosomes residing in the recombining sex, females (2/3 vs. 1/2; Langley et al. 1988; Betancourt et al. 2004). We previously argued that the elevated LD on the autosomes is likely due to positive selection (Garud et al. 2015, 2021). Consistent with previous conclusions based on depressed nucleotide diversity on the X, positive selection may also be responsible for the elevated LD on the X. In the next section, we explore the role of positive selection on the X.

#### Detection of Hard and Soft Sweeps on the X Versus Autosomes in DGRP Data

To assess the role of positive selection on the X versus autosomes, we next applied the haplotype homozygosity statistic H12, which has the ability to detect hard and soft sweeps (Garud et al. 2015, 2021). To apply H12 to genomic data, one must first define a window size in terms of number of single nucleotide polymorphisms (SNPs). In Garud et al. (2015), 401 SNP windows were used on the autosomal DGRP data, where the average length of these windows (∼10 kb) was shown to be large enough to avoid detecting regions of high homozygosity due to random fluctuations in diversity, yet not so large that sweeps cannot be detected. With this window size, sweeps with *s* > 0.05 % can be detected.

An H12 scan with 401 SNP windows on the X chromosome shows substantially reduced signal compared with a scan with the same window size on the autosomes (*P* < 2.2e-16, one-sided Wilcoxon rank sum test; supplementary figs. S7 and S8, Supplementary Material online). Given the elevated LD observed in the data compared with neutral expectations (fig. 4) and previous evidence of positive selection acting on the X of *D. melanogaster* (DuMont and Aquadro 2005; Ávila et al. 2014; Glaser-Schmitt et al. 2021), we discarded the hypothesis of very weak or no selection on the X as an explanation of the lack of signal observed in the 401 SNP window scan. Additionally, as shown in Garud et al. (2015) and our simulations (supplementary fig. S9, Supplementary Material online), H12 has power to detect complete hard sweeps. Thus, it is unlikely that H12 has missed such signatures on the X.

Comparing the distribution of the 401 SNP window size in terms of base pairs, we found that the average window length (bp) is ∼1.5 times larger on the X than on the autosomes (*P* < 2.2e-16, one-sided Wilcoxon rank sum test; supplementary fig. S10, Supplementary Material online). Moreover, the increased recombination rate on the X exacerbates differences in window sizes in terms of centimorgans, as the size of the footprint of selection (bp) decreases with higher recombination, resulting in a stronger and faster LD decay (fig. 4, supplementary fig. S6, Supplementary Material online). Therefore, it is possible that 401 SNP windows are too large to effectively detect selection on the X chromosome.

**Fig. 5.**
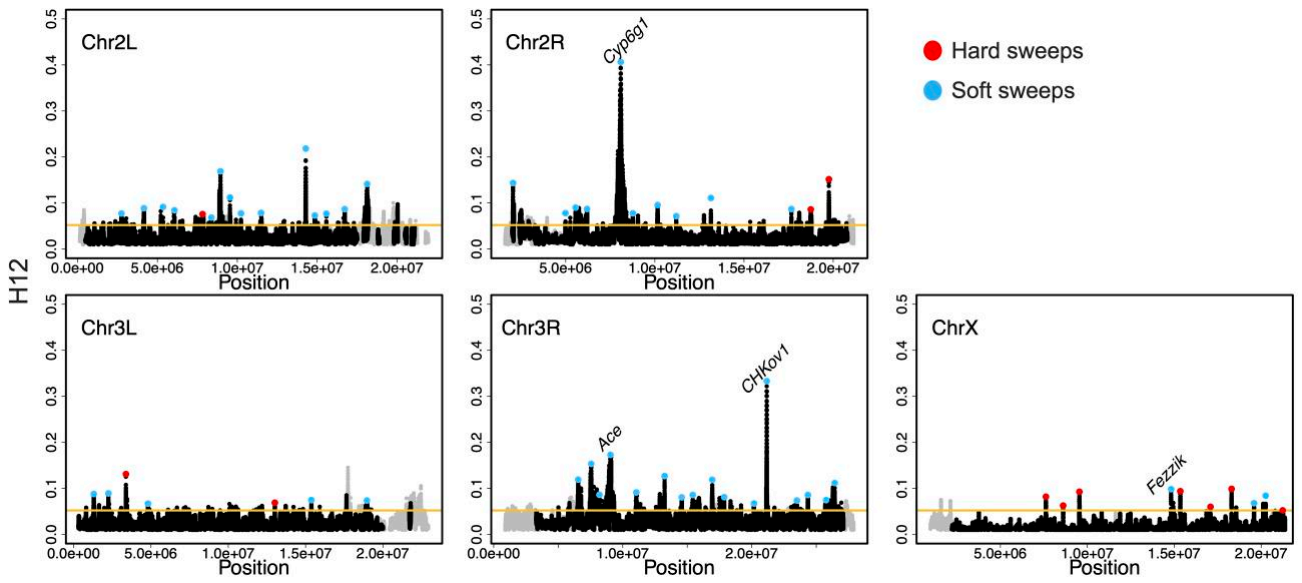
H12 scan in the DGRP data. H12 scan in DGRP data for four autosomal arms and the X chromosome. For the autosomal scan, each data point represents an H12 value in a 401-SNP window down sampled to 265 SNPs. For the X chromosome, windows of 265 SNPs were used. Regions with recombination rates <5 × 10^−7^ cM/bp were excluded from the scan and are denoted in gray. The golden line represents the 1-per-genome FDR line calculated under admixture Model 1 and a recombination rate of 5 × 10^−7^ cM/bp (see Materials and methods). The red and blue data points denote the top 50 and top 10 autosomal and X chromosome peaks, respectively. Blue and red data points correspond to the peaks that were classified as soft and hard sweeps, respectively, by our ABC approach with demographic Model 1, as described in the section “Softness of Sweeps on the X versus Autosomes”. The four positive controls (*Ace*, *Cyp6g1*, *CHkov1*, and *Fezzik*) are highlighted in the scan.

To be able to define H12 analysis windows that are more comparable in terms of base pair length between the X chromosome and the autosomes, we defined smaller windows for the X chromosomes with an average length ∼10 kb (fig. 5). More concretely, we used the autosomal and X chromosome *S*/bp median values obtained from the DGRP data to redefine the number of SNPs per window in our H12 scan. The median *S*/bp in the autosomes of the DGRP data is 0.0345, which means that 401 SNP windows correspond to a window length of *L* = 11, 623 bp. For a recombination rate of 5e-7 cM/bp, sweeps with *s* ≥ 0.05% are likely to generate a signature that extend approximately *L* = 11, 623 bp (*L* ≈ *s*/[log(*N*_*e*_*s*)*ρ*]). The median *S*/bp on the X chromosome is 0.0227; hence, if we took 401 SNP windows as before, the windows in terms of base pairs would be ∼17,665 bp long. Furthermore, for *ρ* = 5e-7 cM/bp and *Ne*_*X*_ = 0.75*N*_*e*Auto_, sweeps with *s∼* 0.1% or greater would be observed in windows of this length. For higher recombination rates, as in the case of the X chromosome (supplementary fig. S6, Supplementary Material online; Betancourt et al. 2004; Pool et al. 2012), only selective sweeps with ∼*s >* 0.1% would be observed. Therefore, to make the H12 analysis windows of the autosomes and the X chromosome more comparable, we defined the X chromosome windows by 0.0227 × *L* ≈ 265 SNPs, where *L* is the autosomal window distance calculated previously. Furthermore, for the autosomes, we took 401 SNP windows, which we randomly down sampled to 265 SNPs, making our analysis windows equivalent in terms of numbers of SNPs.

#### Softness of Sweeps on the X Versus Autosomes

**Fig. 6.**
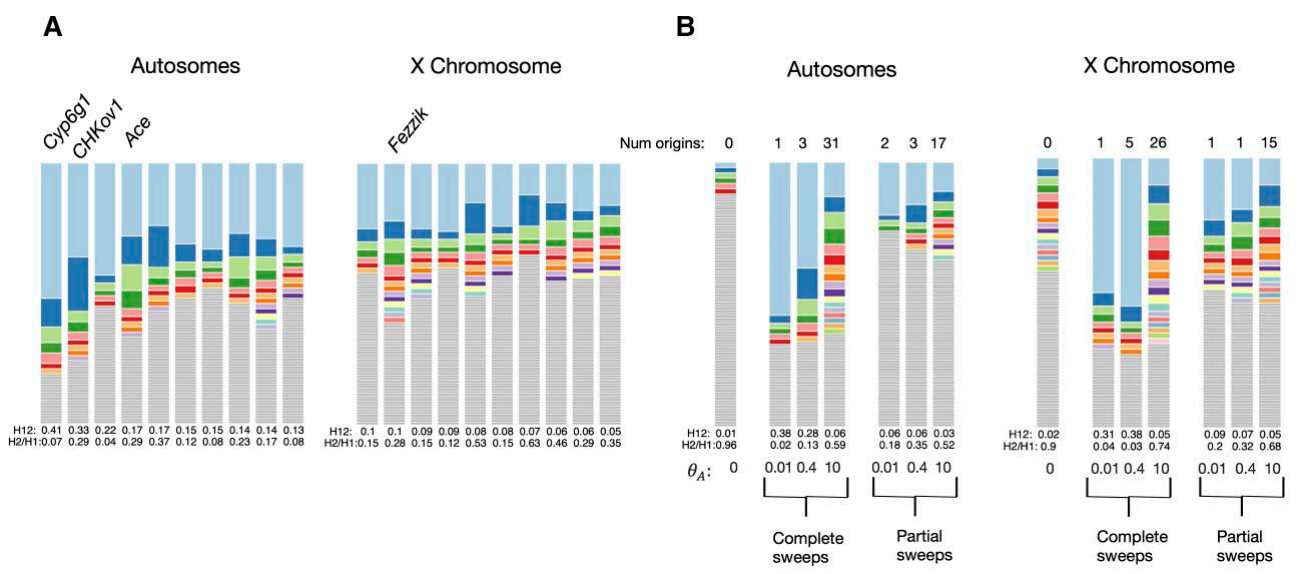
Haplotype frequency spectra for top DGRP peaks and simulated hard and soft sweeps. (*A*) Top 10 autosomal and X chromosome peaks. The analysis window with the highest H12 value is plotted and peaks are ordered from highest to lowest H12 (left to right). Each colored bar represents a distinct haplotype, and the size of the bar corresponds to its frequency in the sample. Gray bars indicate singletons. (*B*) Expected haplotype frequency spectra in simulations of a neutral constant *N_e_* model and selective sweeps from de novo recurrent mutations. These simulations were randomly chosen. Compared are three complete sweeps and three partial sweeps with PF = 0.5. The adaptive mutation rate *θ*_*A*_ is varied to be 0.01, 0.4, and 10 with *h* = 0.5.

To gain intuition on the haplotype structure of the top peaks of the autosomes and the X chromosome, we visualized their haplotype frequency spectra (fig. 6*A*). We also visualized the haplotype frequency spectra of hard and soft, partial, and complete sweeps from simulations (fig. 6*B*). Several peaks on the autosomes have multiple haplotypes at high frequencies, consistent with signatures of soft sweeps, whereas more peaks on the X have haplotype frequency spectra that resemble partial and hard sweeps.

To determine whether the top peaks in our scan were more likely generated by hard or soft sweeps, we computed H2/H1 (Garud et al. 2015), which in conjunction with high H12 values, can differentiate hard and soft sweeps (Garud and Rosenberg 2015; Garud et al. 2015). H2/H1 is the ratio of haplotype homozygosity excluding the most frequent haplotype (H2) and standard haplotype homozygosity (H1). Given that in a hard sweep a single haplotype is found at a high frequency, hard sweeps are expected to have low H2/H1 values. As sweeps become softer, more haplotypes are present at substantial frequencies, increasing H2/H1 monotonically with the softness of the sweep (Garud and Rosenberg 2015). Thus, as proposed by Garud et al. (2015), H12 together with H2/H1 can distinguish whether a sweep is more likely to be hard or soft.

**Fig. 7.**
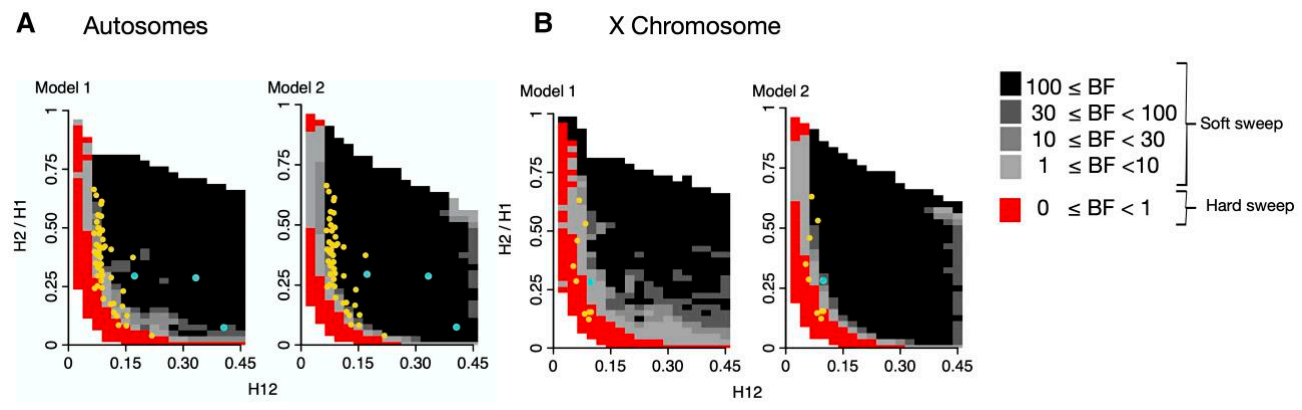
Expected H12 and H2/H1 parameter region for hard and soft sweeps for the autosomes and X chromosome under two variations of the Duchen et al. admixture demographic models. In Model 1, North American *N_e_* = 1,110,000, and in Model 2, *N_e_* = 15,984,500. We obtained the expected H12, H2/H1 parameter region expected for hard and soft sweeps calculating BFs for a grid of H12 and H2/H1 values. We calculated BFs by computing the ratio of soft and hard sweeps obtained from simulations found within a Euclidean distance of 0.1 of an (H12, H2/H1) pair. The (H12, H2/H1) parameter region that is more likely to be generated by hard sweeps is indicated in red (BF < 1). The parameter region that is more likely to represent soft sweeps is shown in gray (BF ≥1), where the darker the gray the higher the likelihood of a soft sweep (BF ≥30). The yellow and blue dots correspond to the top 50 autosomal peaks (*A*) and top 10 X chromosomal peaks (*B*). The blue dots are the positive controls at *Ace*, *CHKov1*, *Cyp6g1*, and *Fezzik*. We used 401 SNP windows down sampled to 265 for the autosome simulations and 265 SNP windows for the X chromosome simulations.

We used an approximate Bayesian computation (ABC) approach to differentiate the likelihood that a given (H12, H2/H1) pair is generated by a hard or a soft sweep model (fig. 7). This likelihood is given by Bayes factors defined as BF = *P*(H12_obs_, H2_obs_/H1_obs_ | soft sweep)/*P*(H12_obs_, H2_obs_/H1_obs_ | hard sweep), where H12_obs_ and H2_obs_/H1_obs_ were computed from the DGRP data. Hard sweeps have BF ≤1, whereas soft sweeps have BF > 1, with stronger evidence for soft sweeps given for BF >> 1.

We simulated hard and soft sweeps under the admixture Models 1 and 2, drawing the selection coefficient (*s*) and start time of selection *T_E_* from uniform prior distributions *s* ∼ *U*[0, 1] and *T*_*E*_ ∼ *U*[0, 10^−3^] × 4*N*_*e*_ (Materials and Methods). Hard sweeps were generated under *θ*_*A*_ = 0.01 and soft sweeps under *θ*_*A*_ = 10 (Pennings and Hermisson 2006a; Hermisson and Pennings 2017). We ran 5 × 10^5^ hard and soft sweep forward-time simulations for each admixture model using the simulator *msms* (Ewing and Hermisson 2010) for a constant recombination rate of *ρ* = 5 × 10^−7^ cM/bp. Given that the average recombination rate is higher on the X than on the autosomes (supplementary fig. S6, Supplementary Material online; Comeron et al. 2012; Pool et al. 2012), we also simulated hard and soft sweeps on the X with *ρ* = 1 × 10^−7^ and *ρ* = 1 × 10^−6^ cM/bp (supplementary fig. S11, Supplementary Material online). With *msms* we are able to run the high number of simulations required for ABC while accounting for demography, which is not computationally feasible using SLiM (see Discussion). Additionally, to be able to include PF after selection ceased (PF∼*U*[0, 1]) and dominance (*h* ∼*U*[0, 1]) as nuisance parameters, as well as male hemizygosity, we performed simulations in SLiM with a constant scaled *N_e_* = 2.7 × 10^6^ model, previously fit to the DGRP data (Garud et al. 2015; see Materials and Methods; supplementary fig. S12, Supplementary Material online). With this simulation approach, we tested the ability of our ABC approach to distinguish simulated hard and soft sweeps (supplementary fig. S13, Supplementary Material online) and found that the majority of sweeps are correctly classified.

The panels in figure 7 and supplementary figures S11 and S12, Supplementary Material online show the BFs calculated from our simulations for a grid of H12 and H2/H1 values. In both Models 1 and 2, we observe a significantly higher proportion of Hard/Soft Sweeps on the X chromosome than on the autosomes (one-tailed exact Fisher's test *P* = 0.001 and *P* = 2.75 × 10^−5^ for Models 1 and 2, respectively). For X chromosome simulations with higher recombination rates, we still observe a significantly higher proportion of hard/soft sweeps compared with autosomes, although the number of sweeps classified as hard decreases when recombination is high (supplementary fig. S11, Supplementary Material online). Additionally, for the constant *N_e_* model, in which PF and *h* were also treated as nuisance parameters, we also observe an enrichment of hard sweeps on the X (one-tailed exact Fisher's test *P* = 0.0001). This suggests an enrichment of hard sweeps on the X chromosome that is robust to demography.

## Discussion

It has been suggested that due to male hemizygosity, the X chromosome experiences more efficient selection as well as an accelerated rate of evolution compared with autosomes (Charlesworth et al. 1987; Betancourt et al. 2004; Singh et al. 2007; Meisel and Connallon 2013; Nam et al. 2015). However, despite widespread interest in the evolutionary forces shaping the X chromosome and the autosomes, our understanding of the tempo and mode of adaptation in natural populations is still forming with the emergence of new data sets (MacKay et al. 2012) and statistical methods to detect selection (Voight et al. 2006; Ferrer-Admetlla et al. 2014; Garud et al. 2015; Schrider and Kern 2016; Sheehan and Song 2016; Kern and Schrider 2018; Szpiech et al. 2021). In this study, we found that sweeps are, on average, expected to be harder on the X than on autosomes in a variety of simulated evolutionary scenarios. Confirming this prediction, we found evidence supporting an enrichment of hard sweeps on the X chromosome of North American *D. melanogaster* in the DGRP data set.

Previous theoretical models from the “Faster-X” literature (Charlesworth et al. 1987; Orr and Betancourt 2001; Betancourt et al. 2004; Meisel and Connallon 2013) show that when adaptation is driven by new mutations, the rate of evolution of recessive mutations is expected to be faster on the X than on the autosomes (supplementary fig. S1, Supplementary Material online; Charlesworth et al. 1987). However, when adaptation proceeds from SGV, Orr and Betancourt (2001) found that the rate of evolution is expected to be slower on the X regardless of dominance, due to a lower probability of fixation and consequently a lower chance of contributing to adaptation (supplementary fig. S4, Supplementary Material online, fig. 2*D*). Confirming this in our simulations, when adaptation proceeds from de novo mutations on the X, we see softer sweeps (e.g., in the case of fig. 1*A* when *h* = 0), but when adaptation proceeds from SGV on the X, we see harder sweeps (fig. 2, supplementary figs. S2 and S3, Supplementary Material online).

Our finding that hard sweeps are enriched on the X in *D. melanogaster* is in accordance with recent work. Recently, Muralidhar and Veller (2022) showed that dominance shifts can lead to softer sweeps on the autosomes. They suggested that because the X is hemizygous in males and thus cannot experience dominance shifts in males, soft sweeps from SGV may be less common on the X, which we confirm. Additionally, recent work in great apes showed that deep dips in diversity on the X chromosome are more consistent with hard sweeps when compared with soft sweeps (Nam et al. 2015), providing empirical support for the notion that hard sweeps may in fact be common on the X. Finally, it has also been suggested that *D. mauritiana*'s X chromosome experiences more hard than soft sweeps (Garrigan et al. 2014).

Consistent with Singh et al. (2007), which found that neutral demography alone cannot account for dips in diversity on the X relative to the ancestral African population and autosomes, we found evidence for selective sweeps on the X. To do so, we extended the autosomal H12 scan we performed in previous work (Garud et al. 2015, 2021), in which we found evidence for abundant soft sweeps.

Due to the significant reduction of diversity on the X chromosome (fig. 4), we defined H12 windows for the X and autosomes such that they are comparable both in terms of length and SNP density. We note that a window defined with a fixed number of base pairs instead of SNPs can potentially result in noisier scans because of random dips in diversity due to drift and background selection. In contrast, defining windows with a fixed number of SNPs ensures that H12 does not co-vary with the number of SNPs available per window. Down sampling the number of SNPs for the autosomal windows from 401 to 265 did not alter the results obtained with the original 401 SNP windows (fig. 5 and supplementary fig. S7, Supplementary Material online), including detection of known soft sweeps at *Ace*, *Cyp6g1*, and *CHKov1.* Moreover, using 265 SNP windows on the X allowed us to recover the signal near *Fezzik*, which was not possible with 401 SNP windows (supplementary fig. S7, Supplementary Material online). In the future, our window approach may be useful for studies comparing populations with substantial differences in their diversity levels.

The recovery of the *Fezzik* locus in our scan was confirmatory given that previous work has shown that the *Fezzik* enhancer has experienced directional positive selection in derived populations of *D. melanogaster* compared with populations from sub-Saharan Africa (Saminadin-Peter et al. 2012; Glaser-Schmitt and Parsch 2018; Glaser-Schmitt et al. 2021). The *Fezzik* gene has been shown to affect tolerance to cold and insecticides (Glaser-Schmitt and Parsch 2018) and is also thought to be involved in ecdysteroid metabolism (Iida et al. 2007) and oxidoreductase activity (Gramates et al. 2017). However, whether this locus experienced a hard or soft sweep was previously unknown. Interestingly, this peak was classified as soft in our ABC analysis. This result may be consistent with the fact that Glaser-Schmitt et al. (2021) showed that an SNP located within the *Fezzik* enhancer is likely under balancing selection as a result of sexually antagonistic forces and temporally fluctuating selection acting on *Fezzik* expression in males and females (Glaser-Schmitt et al. 2021). Their results predict that the variant under selection is likely female beneficial and dominant, although varying dominance might be involved. Consistent with the variant being female beneficial and dominant, our simulations of sexual antagonism (fig. 3) showed that when mutations are beneficial in females and deleterious in males, the likelihood of soft sweeps increases with dominance for both the X and the autosomes.

The *Fezzik* case example provides some biological insight into the underlying mechanisms that could generate soft sweeps on the X. However, being able to accurately distinguish which scenario is driving adaptation is challenging, as the observed signatures could be a result of multiple evolutionary processes. For example, other scenarios that could explain soft sweeps on the X include adaptation occurring by recurrent recessive beneficial mutations. Similarly, as we show in our simulations, hard sweeps on the X could be the result of partially dominant mutations or adaptation to a change in the environment through constant dominance or dominance shifts (figs. 1–3). Additional simulations that incorporate more variations to the model of sexual antagonism such as varying dominance, differences in the magnitude of selection between males and females and temporally fluctuating sex-dependent selection, and variations on demography are needed to better understand the effect of more complicated evolutionary scenarios on the signatures of selection.

We acknowledge that the demographic models used for our ABC analysis are only estimates of the North American *D. melanogaster's* population history and may not fully capture the complexity of this admixed population. To the best of our knowledge, a neutral model that fits the data in terms of *S*/bp, *π*/bp, and long-range LD is not currently available. We therefore used two admixture models proposed in Garud et al. (2021), as these were shown to provide a better fit to the data than previous models (Duchen et al. 2013; Garud et al. 2015; Harris et al. 2018; Arguello et al. 2019). Our results showed evidence for an enrichment of hard sweeps on the X chromosome of the DGRP data regardless of the underlying demographic model tested (fig. 7) or increased recombination rate of the X (supplementary fig. S6, Supplementary Material online). In addition, we tested a constant *N_e_* model with male hemizygosity incorporated and found similar results (supplementary fig. S12, Supplementary Material online). Future work that explores the multidimensional parameter space of *D. melanogaster's* demographic history in search of a model that fits multiple genome-wide statistics to the data would greatly benefit the field. We note that it is possible that to obtain a model that provides a good fit across population genetic statistics, more complex scenarios, such as the effect of seasonal fluctuations (Bergland et al. 2014; Rudman et al. 2022) might need to be included (Johri et al. 2020).

Ideally, our simulations of X chromosome and autosome evolution would simultaneously incorporate both male hemizygosity and admixture. However, doing so is currently a challenge as forward in time simulators such as SLiM (Haller and Messer 2019), that provide the flexibility to model sex chromosome evolution, dominance shifts, and sexual antagonism, cannot simultaneously handle selection and the large effective population sizes and complex demography of *D. melanogaster* populations (Haller and Messer 2019). On the other hand, coalescent simulators like *msms* (Ewing and Hermisson 2010) can model complex admixture events for large *N_e_* but cannot model male hemizygosity. Approaches such as rescaling and tree sequence recording have been shown to increase the computational efficiency of forward in time simulators (Uricchio and Hernandez 2014; Lange and Pool 2018; Haller et al. 2019). Nonetheless, rescaling has not been proved to maintain the genetic diversity of the original model when complex demography and selection are simulated together. Simulating the admixture models in SLiM with the parameters in their original scale would be computationally unfeasible. To address this issue, we modeled the X chromosome by reducing its effective population size to three-fourth of the *N_e_*_Auto_ and ran our admixture models using *msms* (Ewing and Hermisson 2010). Additionally, we ran a constant *N_e_* model in SLiM, incorporating male hemizygosity.

Although we now have evidence for a handful of species in which hard sweeps are more common on the X than the autosomes, it remains to be seen if this is generically true of all species. If hard sweeps are more prevalent on the X than on the autosomes across populations, future work could seek to answer whether hard sweeps are important in driving sexual dimorphism and speciation, where the X chromosome has been shown to play a significant role (Rice 1984; Presgraves 2008; Innocenti and Morrow 2010; Payseur et al. 2018). Moreover, continuing to study the signatures of selection on the X and the autosomes will further increase our understanding on how demographic forces, as well as other evolutionary variables, such as dominance, differentially affect the X and the autosomes.

## Materials and Methods

### Data Processing

We used the publicly available Drosophila Genome Nexus data set (Lack et al. 2015), which includes 205 DGRP strains from Raleigh (RAL), North Carolina and 197 DPGP3 strains from Zambia (ZI). These data can be downloaded at www.johnpool.net.

In order to avoid false positives resulting from IBD from closely related strains, we removed strains with genome-wide IBD levels >20% with at least one other strain. These correspond to 8 ZI strains and 27 RAL strains: ZI397N, ZI530, ZI269, ZI240, ZI218, ZI207, ZI523, ZI86, RAL-385, RAL-358, RAL-712, RAL-399, RAL-879, RAL-355, RAL-810, RAL-350, RAL-832, RAL-882, RAL-306, RAL-799, RAL-801, RAL-859, RAL-907, RAL-790, RAL-748, RAL-336, RAL-850, RAL-365, RAL-786, RAL-730, RAL-861, RAL-59, RAL-646, RAL-812, and RAL-787. This resulted in a total of 178 RAL strains and 189 ZI strains.

The North Carolina DGRP data set consists of data from flies that were extensively inbred to obtain mostly homozygous genomes. Nevertheless, this inbreeding process left tracts of residual heterozygosity, which, in some cases, are substantial. These tracts of residual heterozygosity were treated as missing data, and, if not accounted for, can give false H12 signals. To reduce the inflation of the H12 statistic caused by the remaining IBD and from the masking of heterozygous sites, we down sampled to the top 100 strains with least amount of missing data for each chromosome, separately. Moreover, we required each site to be called in at least 50% of the genomes (supplementary fig. S14, Supplementary Material online).

### Computation of Summary Statistics

To calculate LD, we used the *R*^2^ statistic in sliding windows of 10 kb, iterating by 50 bp. We only considered SNPs with alleles frequencies between 0.05 and 0.95. SNPs with missing data were excluded and at least four individuals at both SNPSs were required to calculate LD. We then smoothed the LD plots as in Garud et al. (2015) by averaging LD values binned in 20 bp windows until 300 base pairs were reached, after which LD values were averaged in windows of 150 base pairs.

We computed *S*/bp and *π*/bp in non-overlapping 10 kb windows for the DGRP data. We estimated the mean levels of *θ*_*S*_ and the corresponding confidence intervals by bootstrapping. We performed 1,000 bootstrap replicates per estimator and constructed the 95% confidence intervals corresponding to each bootstrapped distribution.

### SLiM Simulations

We used SLiM 3.7 (Haller and Messer 2019) to simulate autosomal and X chromosome evolution. For simplicity, we simulated a constant *N_e_* = 10^6^ demographic model under three different scenarios: recurrent beneficial mutations, adaptation from SGV, and sexual antagonism. As SLiM is a forward in time simulator, simulating large population sizes is computationally intensive. We found that for population sizes >5 × 10^5^ simulations become intractable. To make our simulations feasible, we performed rescaling on our model parameters. To do so, we followed Algorithm 1 from Uricchio and Hernandez (2014) with a rescaling constant of *Q* = 50 (Uricchio and Hernandez 2014; Lange and Pool 2018). Both algorithms proposed by these authors closely maintain the levels of genetic variation of the non-rescaled population in a constant *N_e_* model of *D. melanogaster*, as long as selection is not too strong (*s <* 0.1).

In multiple origin sweeps, adaptive mutations arise independently in different individuals; hence, the mutations sweeping through the population have distinct common ancestors. In contrast, in single origin sweeps the adaptive mutation sweeping through the population emerges from a single common ancestor and can recombine onto multiple haplotypes prior to the onset of positive selection thereby resulting in sweeps from SGV. Both can result in either hard or soft sweeps, but we use two different approaches for tracking the softness of a sweep depending on whether the sweep is multi versus single origin. For the multiple origin sweep simulations, SLiM keeps track of mutations that arose independently, hence following (Muralidhar and Veller 2022) we defined sweeps as soft if by the time of fixation, the mutations present in the sweep had distinct mutational origins, and as hard if only one origin was recorded. In the single origin scenario, we cannot define sweeps in a straightforward dichotomous way as before since there is a single label assigned to the beneficial mutation. Therefore, we simulated a 10-kb chromosome and counted the number of distinct haplotypes bearing the adaptive allele at the time of fixation. We used the number of distinct haplotypes as a proxy for the softness of the sweep, where softer sweeps are expected to have a higher number of distinct haplotypes than hard sweeps.

Throughout our simulations we assumed dosage compensation that is mutations on the X of males experienced the same effect on fitness as a female homozygous for the same mutation. Moreover, in all simulations we used a recombination rate of *ρ* = 5 × 10^−7^ cM/bp and neutral mutation rate of *µ* = 1 × 10^−9^ rescaled by *Q* = 50.

The specific simulation set up for each of the three scenarios modeled is described in the following section.

### Recurrent Beneficial Mutations Multiple Origin Sweeps

We started the simulation by introducing a beneficial mutation to the population according to *θ*_*A*_ = 4*N*_*e*_*μ*. We ran simulations for *θ*_*A*_ = 0.04, 0.4, and 4, dominance coefficient *h* = 0, 0.25, 0.5, 0.75, and 1, and selection coefficient *s* such that *N_e_s* = 100. We simulated complete sweeps and recorded the number of distinct mutational origins present at fixation in a sample of 100 haplotypes. We then classified a sweep as hard if only one mutational origin was present in the sample and soft if two or more origins were present. For each parameter combination, a total of 1,000 simulations were run.

### Adaptation from SGV, Constant Dominance, and Dominance Shifts

#### Single Origin Sweeps

We simulated single origin selective sweeps by introducing a single deleterious mutation with *N_e_s_d_* = 0 and *N_e_s_d_* = −100 at the center of a 10-kb chromosome with per locus neutral mutation rate defined by *θ* = 0.004. We tracked the deleterious mutation until it reached a specific PF 0.0001, 0.001, and 0.005, where higher PFs are expected to give rise to softer sweeps, as the mutation spends more time segregating on distinct genetic backgrounds before the onset of positive selection. Once PF was reached, we set the selection coefficient to *N_e_s_b_* = 100 and dominance to *h_b_*.

We simulated a model with constant dominance before and after the change in selection as well as a model of dominance shifts where we set the dominance to be *h_d_* when the mutation is deleterious and *h_b_* = 1 − *h_d_* when the mutation becomes beneficial. We ran the simulation until the sweep fixed or was lost. For those simulations in which the sweep fixed, we computed the number of distinct haplotypes bearing the adaptive allele in each simulation. For each parameter combination, we ran a total of 2,000 fixed sweep simulations.

#### Multiple Origin Sweeps

For the multiple origin model of adaptation to a new environment from the standing variation (fig. 2), we followed the approach described in Muralidhar and Veller (2022). A deleterious mutation with *N_e_s_d_* ≤ 0 and dominance coefficient *h_d_* was introduced to the population with a mutation rate defined by *θ*_del_ = 0.04, 0.4, and 4. We ran the simulation for 10 *N_e_* generations after which we computed the number of mutational origins in the population. If no mutations were present at this time, we stopped the simulation and labeled it as “no SGV.” If mutations were present in the population after the 10 *N_e_* generations, we set the selection coefficient to *s_b_ = − s_d_* and dominance to *h_b._.* After the change in selection, we stopped introducing the recurrent mutation and tracked the presence of the mutations each generation. We ended the simulation if all mutations were lost, labeling this as “lost sweep,” otherwise we ran the sweep until fixation, obtained a sample of 100 individuals and recorded the number of distinct mutational origins in the sweep.

For each set of parameter values (*s_d_*, *h_d_*, *s_b_*, *h_b_*, *θ*_del_ ), we ran a total of 2,000 simulations and simulated (1) a model of constant dominance across environments (*h_b_* = *h_d_*), (2) a model of dominance shifts where *h_b_* = 1 − *h_d_* (fig. 2), (3) a multiple origin sweep model of dominance shifts with *s_d_* = 0 and *N_e_s_b_* = 100 (supplementary fig. S3, Supplementary Material online), as well as (4) a model of dominance shifts in which either *h_b_* or *h_d_* were fixed (supplementary fig. S4, Supplementary Material online).

For the dominance shifts model, we included the scenario in which mutations kept entering the population even after the environmental change, at the same rate as before the shift (supplementary fig. S5, Supplementary Material online). We computed the probability that a sweep contained adaptive mutations that originated from SGV, and in such cases, we obtained the proportion of mutations derived from before and after the shift.

### Sexual Antagonism

We simulated sexual antagonism in two ways: male advantage with female disadvantage and male disadvantage with female advantage. As in the recurrent mutation simulations, we began by introducing a mutation at a rate defined by *θ*_*A*_, for *θ*_*A*_ = 0.04, 0.4, and 4. We set the selection to be sex dependent such that *N_e_s_b_* = 100 in the sex with the adaptive advantage and *N_e_s_d_* = −10 in the sex with the deleterious effect. We ran the simulation until the sweep fixed and recorded the number of distinct mutational origins in the sweep. Again, we defined the sweep as hard or soft if one or multiple origins were present in the sample, respectively.

#### H12 Scan

We ran a genome-wide scan using sliding windows of 401 SNPs down sampled to 256 SNPs for the autosomes and 256 SNPs for the X chromosome, iterating by intervals of one SNP between window centers. To avoid false peaks driven by high missingness, for each analysis window, haplotypes with >10% of missing data were assigned a frequency of 1/*N*, where *N* is the sample size (*N* = 100).

We called peaks by identifying the windows with H12 values above the H12_o_ value, defined as the false discovery rate (FDR) which we took as the 10th highest H12 value obtained from 10 times the number of independent analysis windows in the data (∼100,000) neutral simulations. We obtained H12_o_ for each admixture model as well as a constant *N_e_* = 2.7 × 10^6^ model and chose the highest H12_o_ as our critical value (H12_o_ = 0.052).

We grouped the consecutive windows above the threshold into a single peak and assigned the highest H12 value the value of the peak. We then iterated through the identified peaks, from highest to lowest, and excluded the peaks found within 500 kb of the center of the peak under inspection. This avoided the identification of peaks belonging to the same selective event. We finally masked peaks found in regions of low recombination (<5 × 10^−7^ cM/bp) identified using the Comeron et al. (2012) crossover map.

#### ABC to Classify Hard and Soft Sweeps

We used ABC to compute the likelihood that a soft or a hard sweep model generates a pair of (H12, H2/H1) values. We simulated hard sweeps with *θ*_*A*_ = 0.01 and soft sweeps with *θ*_*A*_ = 10. To be able to run a large number of simulations, we used the coalescent simulator *msms* (Ewing and Hermisson 2010). We ran a total of 5 × 10^5^ simulations for both the hard and soft sweep models for the two admixture models proposed by Garud et al. (2021). These models are variations of the Duchen et al. (2013) admixture model and were fitted to the autosomal DGRP data in terms of the summary statistics *S*/bp, *π*/bp, and H12 while accounting for admixture events in North American *D. melanogaster.* Because *msms* does not have the option to simulate sex chromosome evolution, we simulated the X chromosome by downscaling the effective population sizes by three-fourth.

We drew the values of the nuisance parameters selection strength (*s*) and start time of selection (*T_E_*) from the following prior distributions: *s* ∼ *U*[0, 1] and *T*_*E*_ ∼ *U*[0, 10^−3^] × 4*N*_*e*_. We then calculated Bayes factors for a grid of (H12, H2/H1) values by taking the ratio of the number of soft sweep and hard sweep simulations with a Euclidean distance <0.1 from each (H12, H2/H1) data point from our H12-H2/H1 grid, as done in Garud and Petrov (2016) and Garud et al. (2015, 2021).

Additionally, to incorporate male hemizygosity into our X chromosome simulations, we modeled a constant *N_e_* = 2.7 × 10^6^ population on SLiM. As before, hard sweeps were simulated under *θ*_*A*_ = 0.01 and soft sweeps under *θ*_*A*_ = 10. Due to the large *N_e_* of the model, we downscaled our simulation parameters by a factor of *Q* = 50 (Uricchio and Hernandez 2014) and used tree sequence recording to add neutral mutations to our model and perform recapitation with *msprime* (Kelleher et al. 2016) and *pyslim* (Haller et al. 2019), making the simulations computationally tractable. In addition to the hyperparameters *s* and *T*_*E*_, these simulations include PF after selection ceased (PF ∼*U*[0, 1]) and dominance (*h* ∼*U*[0, 1]).

#### Code Availability

Code used to process and analyze the data is available at: https://github.com/garudlab/SelectiveSweeps_Xchr_vs_Auto.

## Supplementary Material

msac268_Supplementary_Data
