# Supplementary material for: Enrichment of Hard Sweeps on the X Chromosome in Drosophila melanogaster
Source: Mol Biol Evol. 2022 Dec 21;40(1):msac268. doi: 10.1093/molbev/msac268 (PMC9825254; doi:10.1093/molbev/msac268)
Supplement: msac268_Supplementary_Data [file msac268_supplementary_data.pdf]

Supplementary Material

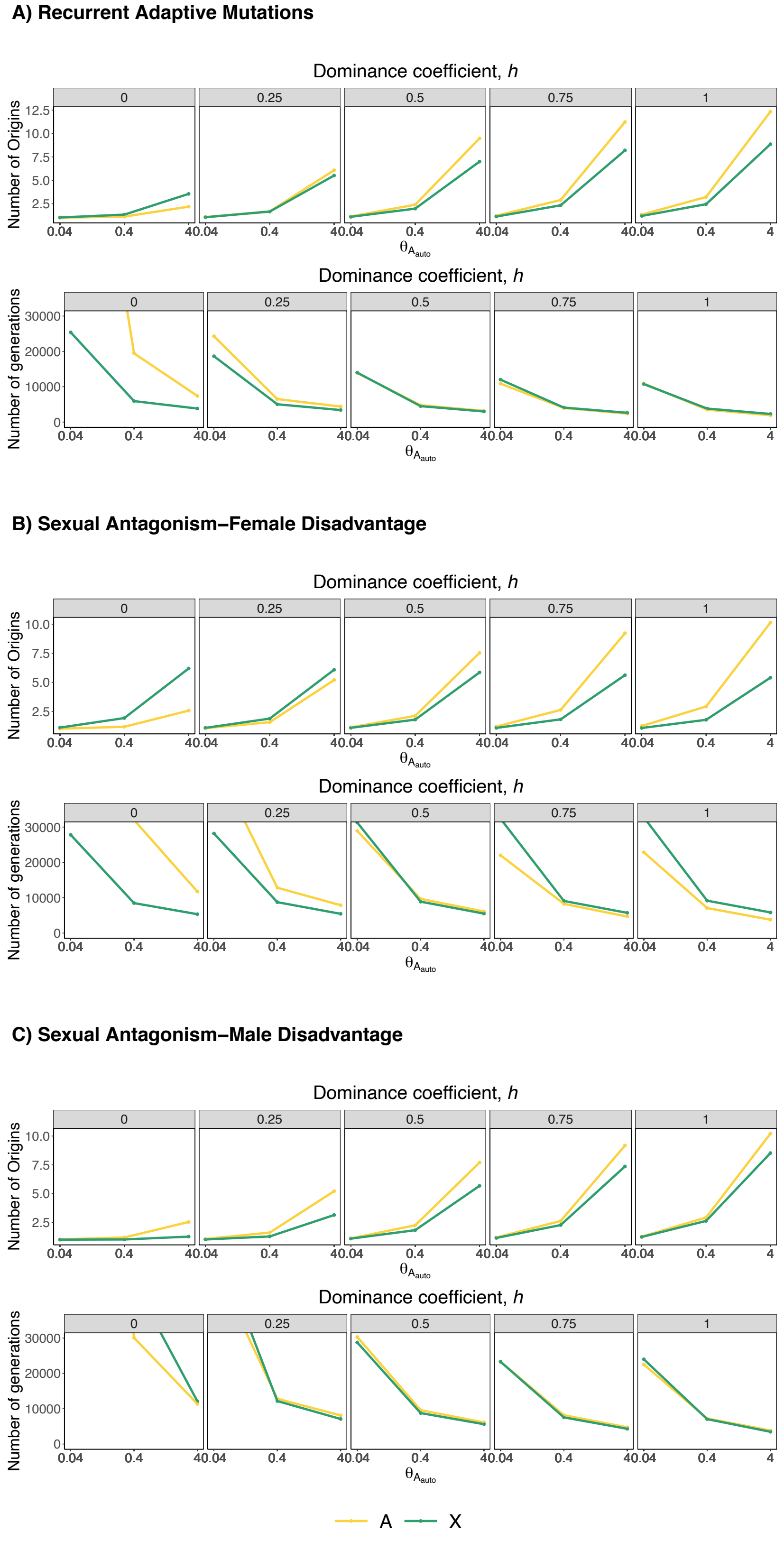

Figure S1. Average number of origins and average number of generations until fixation in three simulated scenarios. 1,000 simulations of each evolutionary scenario per combination of parameters were performed. The scenarios depicted in the figure include (A) Adaptation through recurrent *de novo* mutations, (B) Sexual antagonism with female disadvantage and (C) Sexual antagonism with male disadvantage.

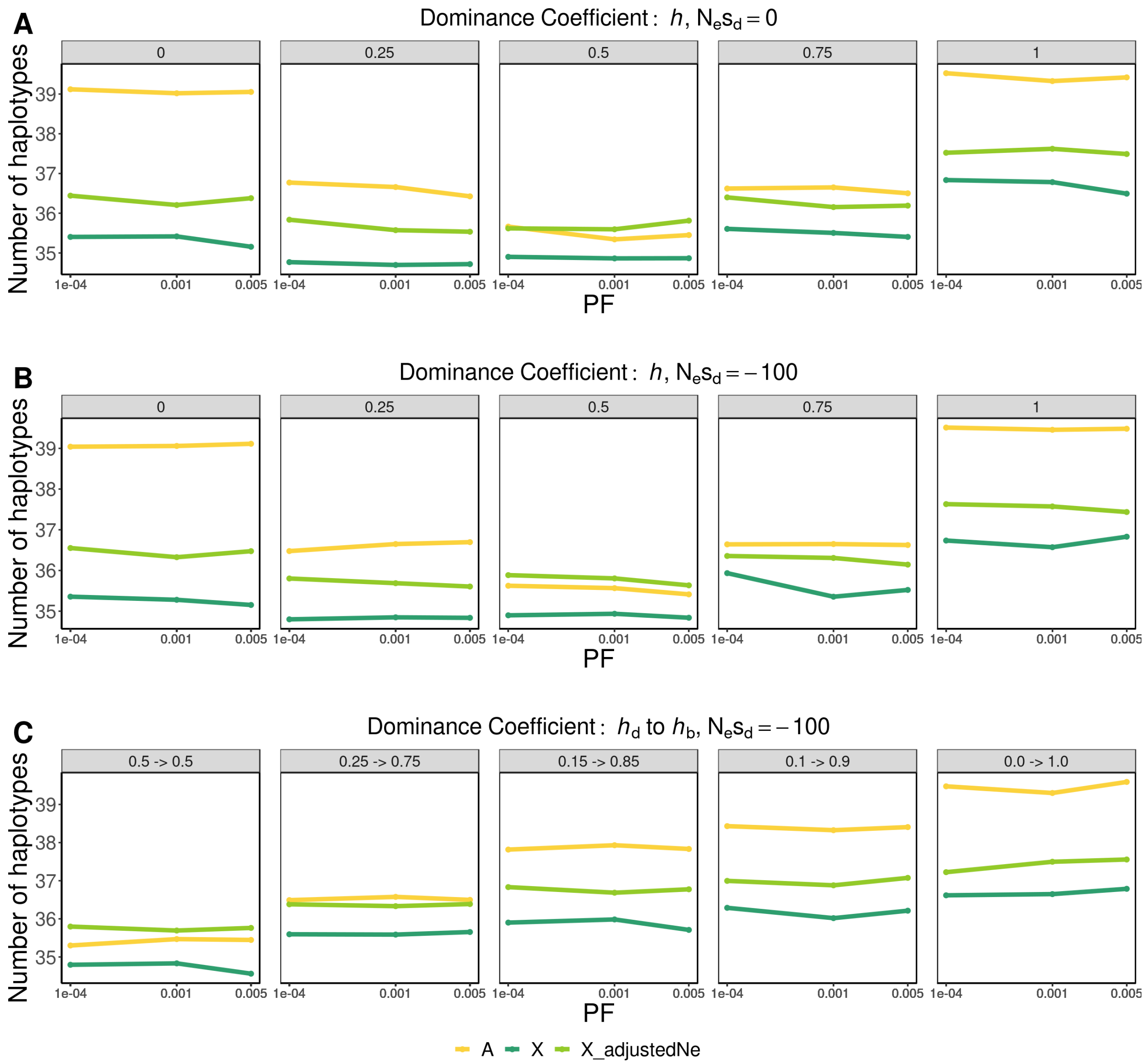

Figure S2. Number of distinct haplotypes in simulations of sweeps from SGV. We modeled a sweep from SGV arising from a single origin, where an adaptive mutation sweeping through the population emerges from a single common ancestor. To do so we simulated a 10kb chromosome and introduced a single neutral **(A)** or deleterious mutation with $N_e s_d$ =-100 **(B,C)** at the center of the chromosome. We tracked the frequency of the mutation in the population until a given partial frequency (*PF*) was reached (specified on the X-axis of the plots above). After this the mutation became beneficial and the simulation ran until fixation. The average number of distinct haplotypes were computed across 2,000 selective sweep simulations with constant dominance

(A,B) and dominance shifts **(C)**. We considered the number of distinct haplotypes to be a proxy for the softness of the sweep, where soft sweeps are expected to have a higher number of distinct haplotypes than hard sweep. The yellow lines correspond to the autosome and the green lines to the X chromosome. To test the effect of population size versus hemizygosity on the softness of a sweep, we adjusted the N_e of the X in light green to match that of autosomes. The X chromosome sweeps have a lower number of distinct haplotypes than the autosomes, suggesting that sweeps on the X are harder. This difference is greatest when a mutation is completely recessive or completely dominant and when the shift in dominance is strongest.

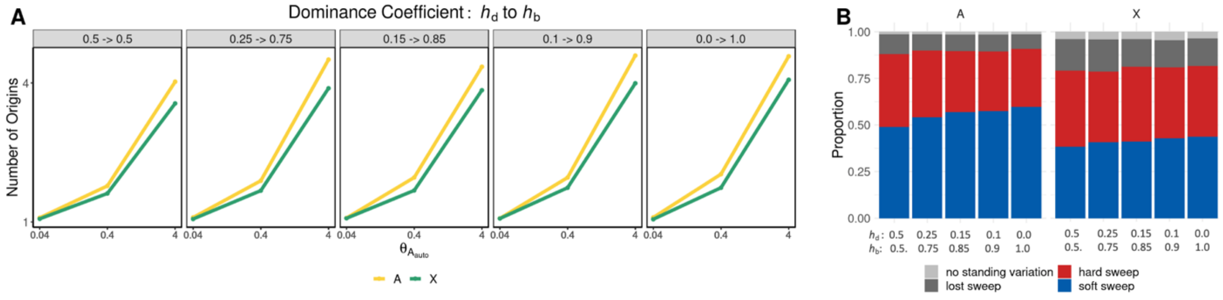

Figure S*3*. Dominance shifts with s_d=0. The number of origins in X and autosome simulations for θ_A= 0.04, 0.4 and 4 in a dominance shifts model where a neutral mutation (s_d=0) is initially segregating in the population **(A)**. **(B)** The proportion of hard and soft selective sweeps in a dominance shifts model for $\theta_{\mathrm{del}_{\mathrm{auto}}} = 0.4$. For each combination of parameters, we ran a total of 2,000 simulations of a constant scaled N_{eAuto}=1e6 model, with s_d and $N_e s_b$ =100, $\theta_{\mathrm{del}_{\mathrm{auto}}} = 0.4$, and $\theta_{\mathrm{del}_X} = 0.75\theta_{\mathrm{del}_{auto}}$.

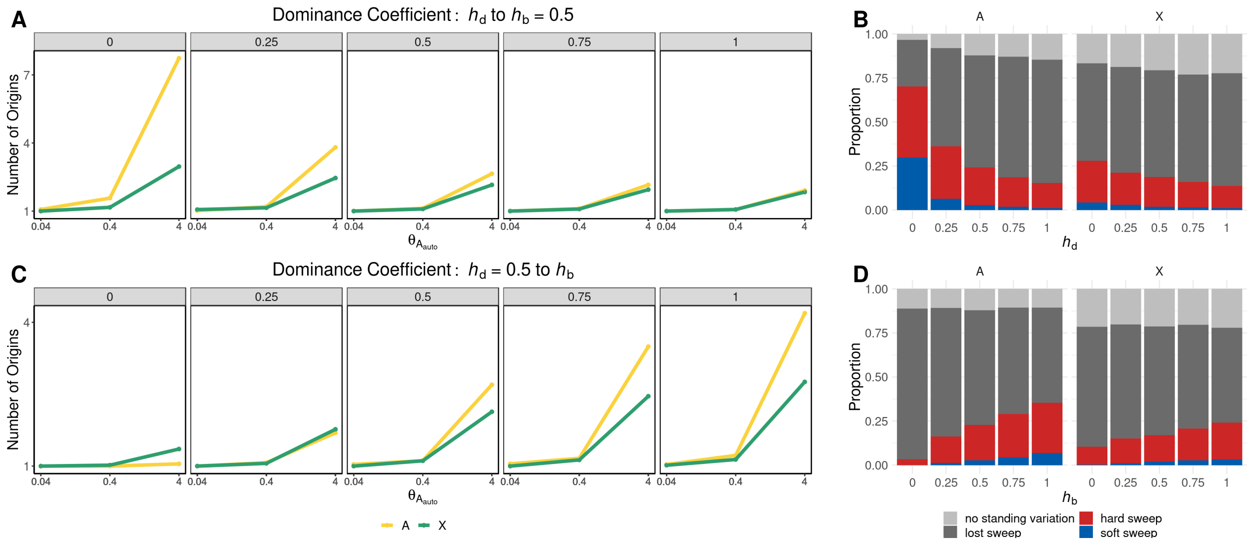

Figure S4. Effect of dominance on the softness of a sweep. The number of origins on the autosomes and the X for simulations performed with θ_A= 0.04, 0.4 and 4. In **(A)** h_b =0.5 and h_d is varied as specified in each plot. Likewise, in **(C),** h_d =0.5 and h_b is varied. **(B)** and **(D)** show the proportion of hard and soft selective sweeps on the autosomes and the X from the scenarios in (**A**) and (**C**), respectively**.** For each combination of parameters, we ran a total of 2,000 simulations with a constant scaled N_{eAuto}=1e6 model, with $N_e s_d$ and $N_e s_b$ =100, $\theta_{\mathrm{del}_{auto}} = 0.4$, and $\theta_{\mathrm{del}_X} = 0.75\theta_{\mathrm{del}_{auto}}$.

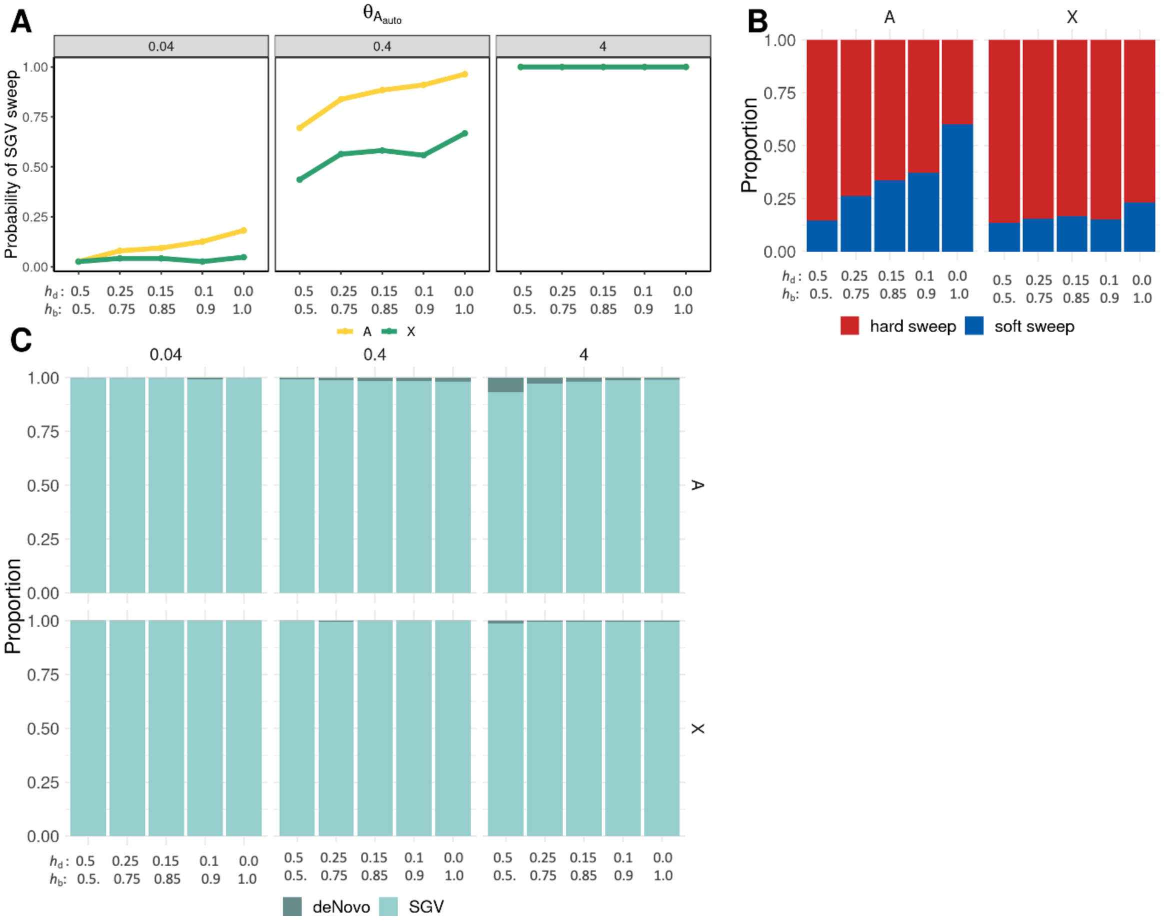

Figure S5. Selective sweeps from SGV with continued input of new mutations after the environmental shift. For 10Ne generation, mutations were permitted to enter the population at the rate of $\theta_{A_{auto}}$, as specified in the grey bar above each plot in (**A**). Any mutations present at the time of the environmental shift became beneficial. Beneficial mutations continued to enter the population with the same $\theta_{A_{auto}}$. We computed the probability that at least one sweeping haplotype bears an SGV allele that arose prior to the environmental shift **(A)** as well as the proportion of hard (red) and soft (blue) sweeps on the autosomes and the X chromosome for $\theta_{A_{auto}}$= 0.4 **(B)**. We also obtained the proportion of sweeps containing alleles from SGV versus exclusively *de novo* mutations for the case $\theta_{A_{auto}}$ = 0.04,0.4 and 4 **(C)**.

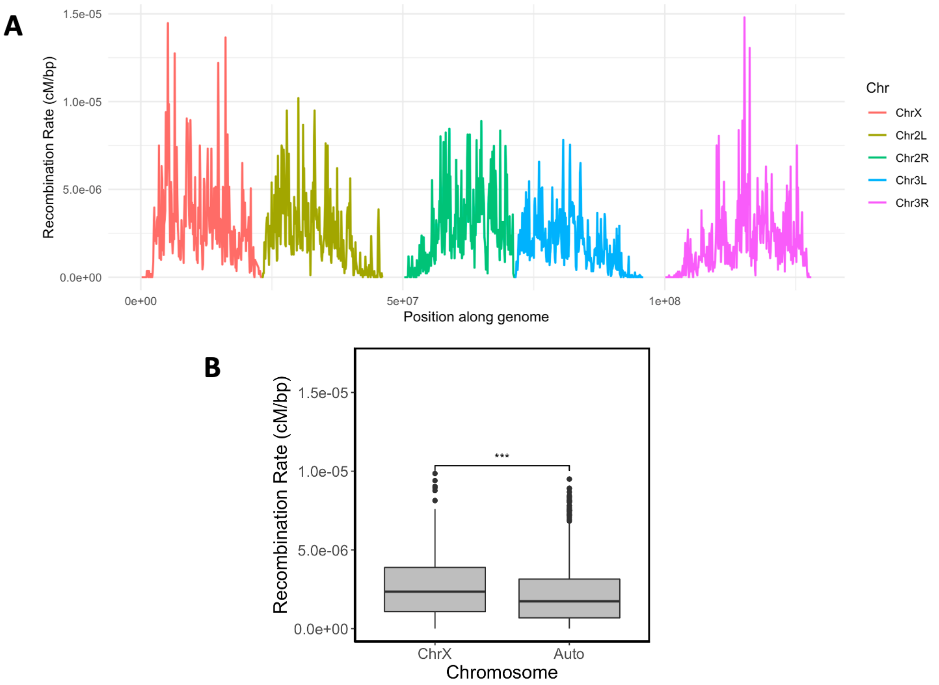

Figure S6. Recombination rates along the genome of *D. melanogaster*, using the Comeron et al. (2012) crossover map. (A) Recombination rates along the genome colored by chromosome. (B) Distribution of X chromosome and autosomal recombination rates. The distributions of recombination rates on the X chromosome is significantly higher than on that of the autosomes (one-sided Wilcoxon rank sum test p-val =8.685e-06).

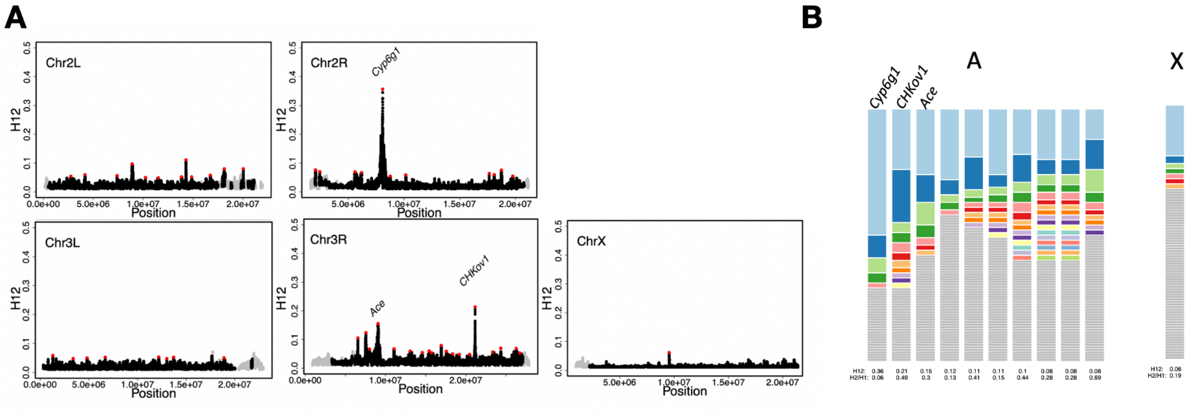

Figure S7. **H12 scan with 401 SNP windows for four autosomal arms and the X chromosome.** (**A**) H12 scan in DGRP data for four autosomal arms and the X chromosome using 401 SNP windows. Gray points correspond to genomic regions with recombination rates lower than 5x10-7 cM/bp. The red data points denote the top 50 autosomal peaks and the only X chromosome peak detected in the 401 SNP window scan. (**B**) Haplotype frequency spectra for the top 10 autosomal peaks and the X chromosome peak. Each colored bar represents a distinct haplotype, and the size of the bar corresponds to its frequency in the sample. Gray bars indicate singletons. The three autosomal positive controls, Ace, Cyp6g1, CHkov1 are highlighted in the figure.

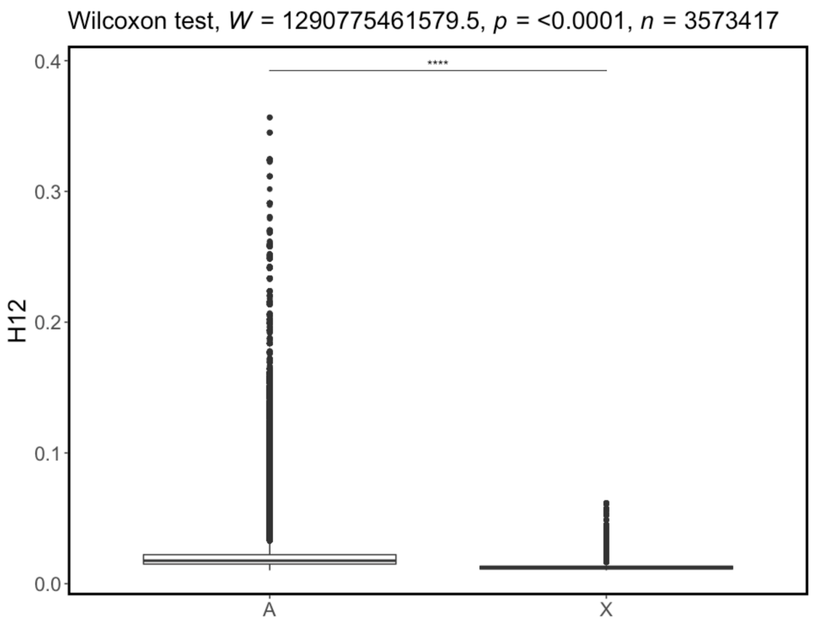

Figure S8. Distribution of H12 values for 401 SNP windows in the autosomes and X chromosome of *D. melanogaster*. A Wilcoxon rank sum test on the distribution of the autosomal and X chromosome H12 values shows that the distributions are significantly different with p-val<2.2e-16.

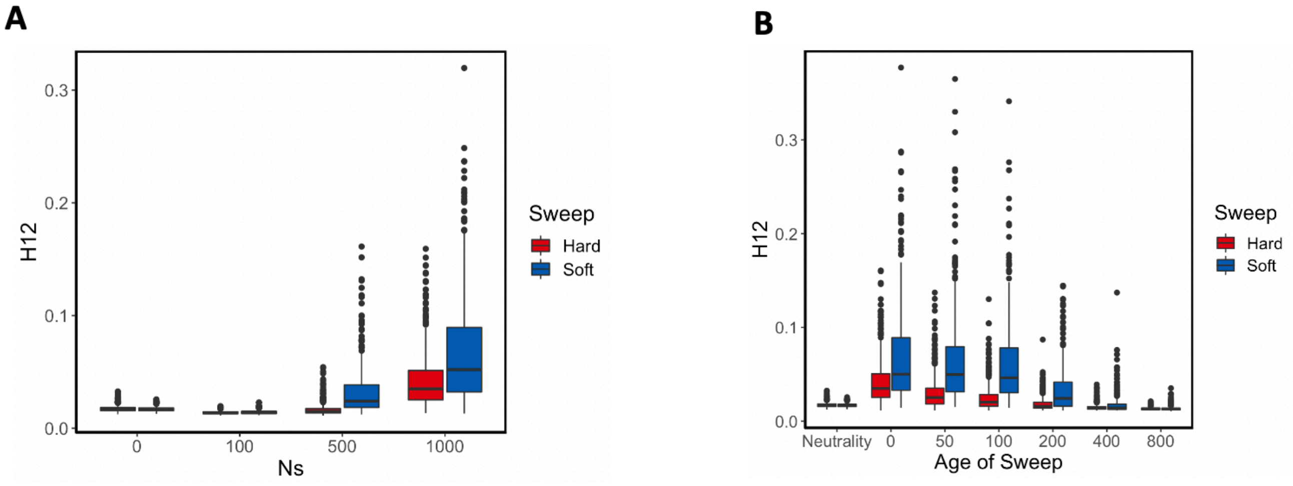

Figure S9. Ability of H12 to detect complete hard and soft selective sweeps. Simulations of complete hard and soft sweeps for varying selection strengths ($N_e s$) and increasing age of the sweep. The age of the sweep is given in number of generations after fixation, when the selection strength ceased. (**A**) Distribution of H12 values for hard (red) and soft (blue) selective sweeps for varying strengths of selection s at the time of fixation. H12 can distinguish selection from neutrality when selection is sufficiently strong. (**B**) Distribution of H12 values for hard and soft sweeps with s=0.1, for increasing number of generations after fixation. H12 can detect selection for sweeps that are not too old (age <200 generations).

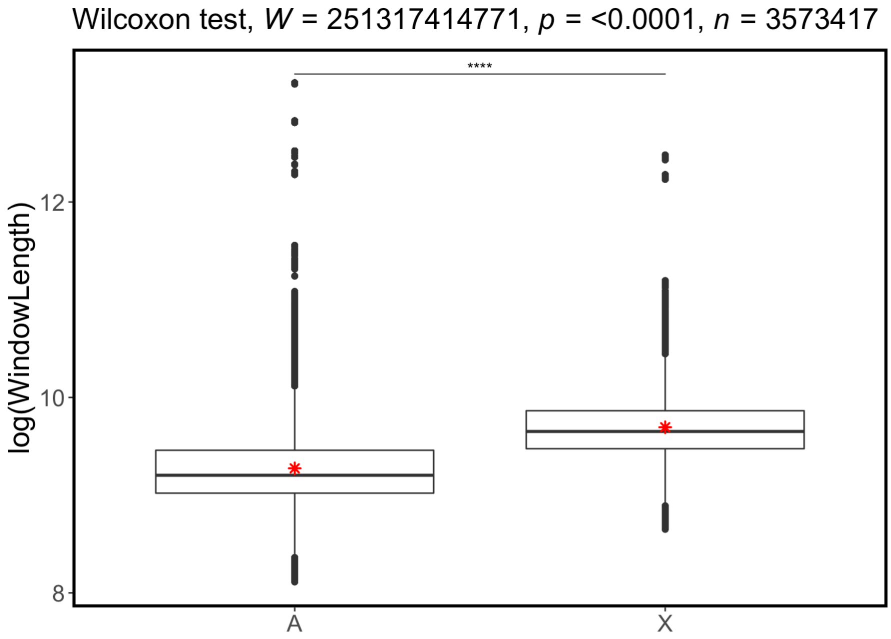

Figure S10. Window length (bp) distributions in H12 scan performed with 401 SNP windows. Distribution of the log of the window lengths for the autosomes (A) and the X chromosome (X). The red star indicates the mean window length in the autosomes and the X, respectively. Autosomes have significantly lower window lengths than the X chromosome in the 401 SNP window scan (p-val<2.2e-16, one-sided Wilcoxon rank sum test).

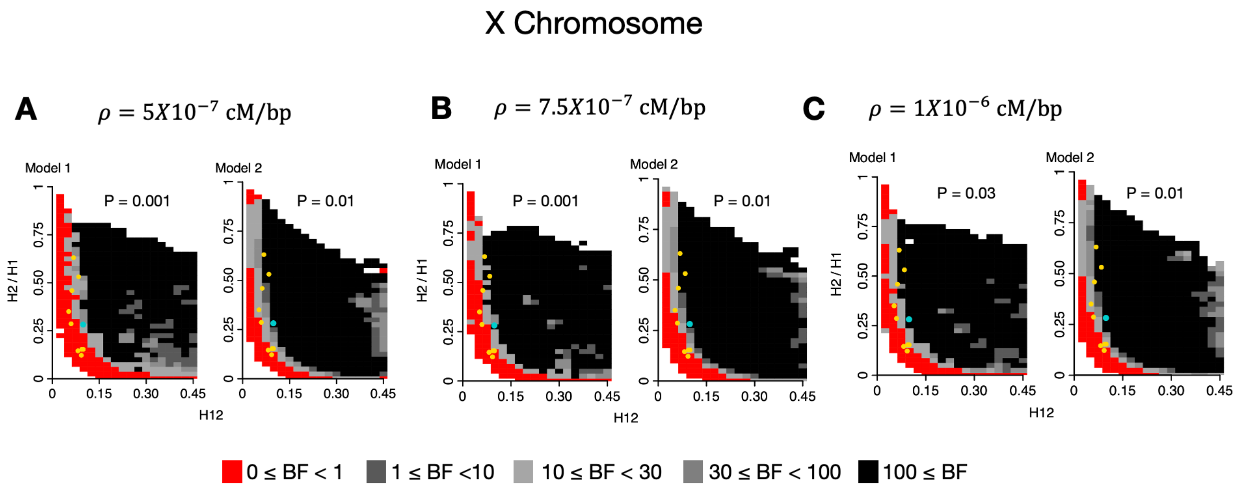

Fig S11. Expected H12 and H2/H1 parameter region for hard and soft sweeps for the X chromosome for varying recombination rates. A total of 10^5 hard and soft sweep simulations were performed for the following recombination rates: ρ = 5x10^{-7} cM/bp (**A**), 7.5x10^{-7} cM/bp (**B**) and 1x10^{-6} cM/bp (**C**). Regions shown in red provide support for hard sweeps whereas regions in gray show support for soft sweeps. Each panel shows a p-value corresponding a one-sided exact fisher test comparing the hard/soft sweep between the X chromosome (scenarios **A-C**) and the autosomes. The number of hard and soft sweeps on the autosomes were obtained from an ABC analysis using ρ = 5x10^{-7} cM/bp (**Figure 7**). These simulations were done on *msms,* accounting for admixture and setting N_{eX}=3/4N_{eAuto}.

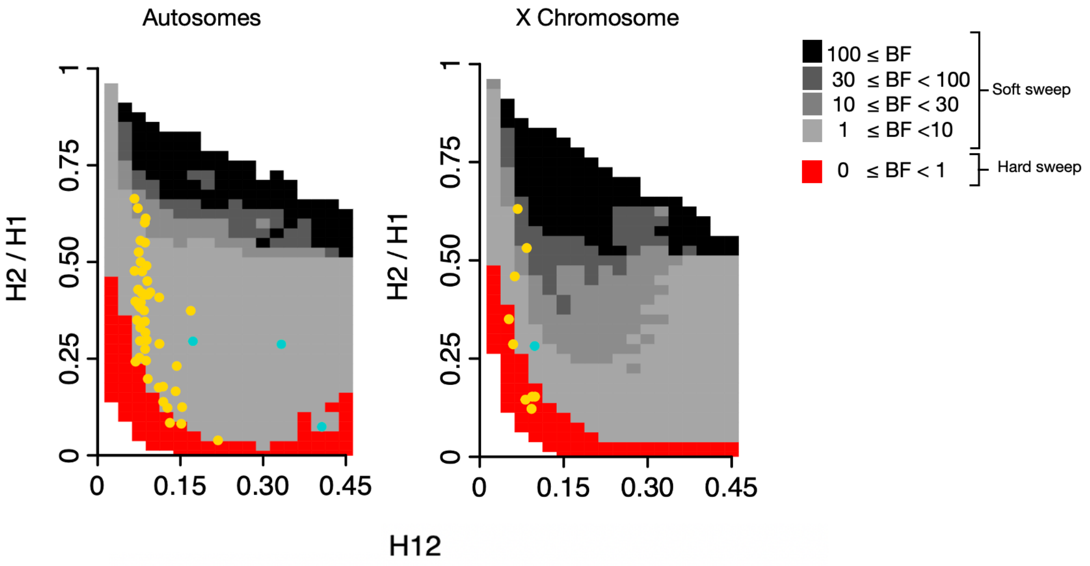

Fig S12. Expected H12 and H2/H1 parameter region for hard and soft sweeps for the X chromosome and the autosomes in a Constant N_e=2.7x10^6 model. ABC analysis to classify H12 peaks as hard and soft. A total of 10^5 hard and soft sweep simulations were performed using SLiM for each the autosomes and the X chromosome. The regions colored in red show parameter values that are more likely to be generated by soft sweeps, while gray regions correspond to the parameter space that is more likely to be generated by soft sweeps. The points highlighted in the two panels correspond to the top 10 H12 peaks on the X chromosome of the DGRP data with the blue point corresponding to the positive controls. Nuisance parameters were drawn from uniform prior distributions as follows: $s \sim U[0,1]$ and $T_E \sim U[0, 10^{-3}] \times 4N_e$, $PF \sim U[0,1]$ and $h \sim U[0,1]$. These simulations were performed on SLiM, modelling the hemizygosity of the X chromosome.

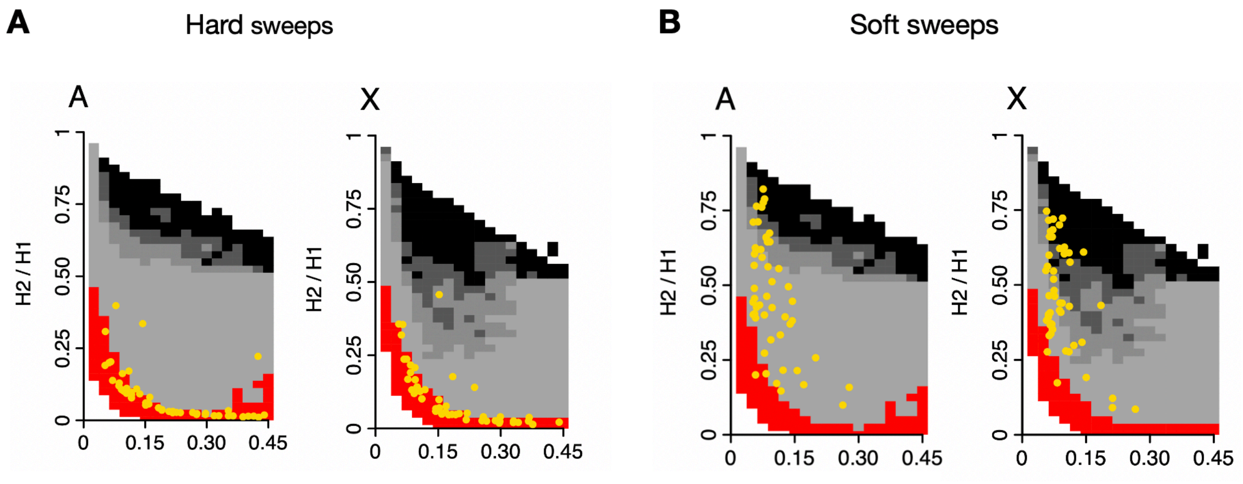

Fig S13. Performance of ABC approach. H12-H2/H1 pairs of 50 hard **(A)** and 50 soft sweeps **(B)** obtained from the constant N_e=2.7x10^6 simulations. These values are given by the yellow data points, where those lying on the red parameter space are classified as hard and those on the gray region are classified as soft. We observe 2-5 false positives depending on the mode of selection and chromosome being simulated, but overall, the majority of the sweeps are correctly classified.

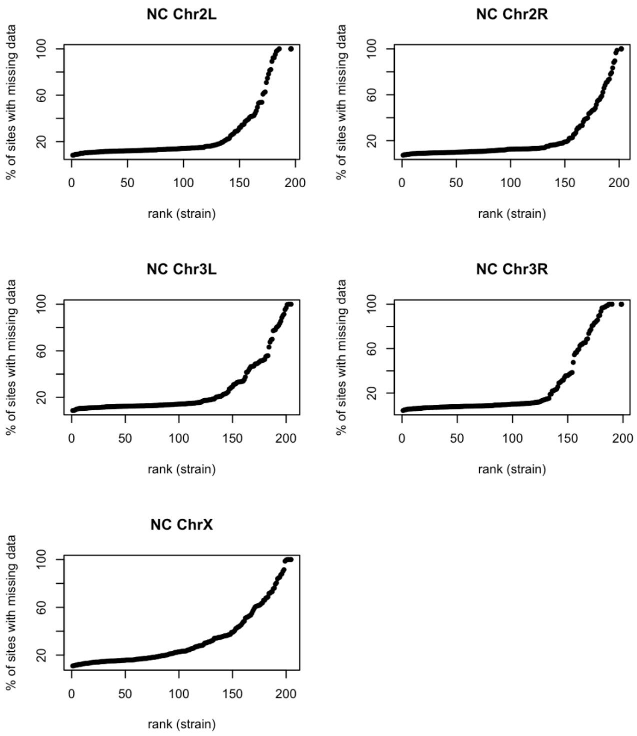

Figure S14. Number of sites with missing data in each strain in DGRP data

Table S1. Demographic parameters for Admixture models from Garud et al. 2021

Shown are the parameter estimates for the admixture models inferred in (Duchen et al. 2013; Garud et al. 2021). The parameters used for both models are explained below.

N_{Aa}	Ancestral African population size
sev_A	Severity of African bottleneck (log_{10} scale duration/population size)
N_{Ac}	Contemporary African population size
N_E	European population size
N_{Am}	North American population size
T_A	Time of bottleneck in Africa (log_{10} scale)
T_{AE}	Time of split between African and European populations (log_{10} scale)
T_{adm}	Time of admixture between Africa and Europe (log_{10} scale)
$prop_{adm}$	Proportion of European admixture

Model 1:

N_{Aa}	sev_A	N_{Ac}	N_E	N_{Am}	T_A	T_{AE}	T_{adm}	$prop_{adm}$
5,224,100	0.21	4,975,360	700,000	1.11e6	5.38	5.29	3.16	0.85

Model 2:

N_{Aa}	sev_A	N_{Ac}	N_E	N_{Am}	T_A	T_{AE}	T_{adm}	$prop_{adm}$
5,224,100	0.21	4,975,360	700,000	15,984,500	5.38	5.29	3.16	0.85
